# Dissecting the host determinants of orthoflavivirus infection using QIC-seq

**DOI:** 10.1371/journal.ppat.1014279

**Published:** 2026-06-26

**Authors:** Allison J. Dupzyk, Benjamin S. Waldman, James Zengel, Fabio Zanini, Jan E. Carette

**Affiliations:** 1 Department of Microbiology and Immunology, Stanford University School of Medicine, Stanford, California, United States of America; 2 School of Clinical Medicine, University of New South Wales, Sydney, New South Wales, Australia; 3 Evolution and Ecology Research Centre, University of New South Wales Sydney, Sydney, New South Wales, Australia; University of California Davis, UNITED STATES OF AMERICA

## Abstract

Orthoflaviviruses are genetically related, yet cause distinct disease patterns ranging from hepatitis and vascular shock syndrome to encephalitis and congenital abnormalities. There is an incomplete understanding of the cellular pathways co-opted by orthoflaviviruses, and differences in host response to infection may underlie the diverse pathologies caused. We present a single-cell approach (Quantification of Infection and CRISPR guide sequencing; QIC-seq) that combines CRISPR/Cas9 knockout with virus-inclusive transcriptomics to systematically compare host factor requirements and host transcriptional response to orthoflaviviral challenge. Using a CRISPR library focused on select ER-proteostasis genes, we show that dengue and yellow fever viruses are strictly dependent on subunits of the oligosaccharyltransferase complex, while the more distantly related West Nile and Langat viruses are dependent on components of the ER-associated degradation machinery. Our data further shows virus-induced upregulation of interferon-stimulated genes, and activation of the unfolded protein response. QIC-seq enables quantitative comparisons of viral host factor utilization, which may inform development of host-directed antiviral therapies.

## Author summary

Divergent Orthoflaviviruses have distinct transmission modes and pathologies, yet share common replication strategies. To better understand host factor requirements for this diverse group of viruses, we have developed a single cell RNA-sequencing strategy called Quantification of Infection and CRISPR guide sequencing (QIC-seq), which simultaneously captures flavivirus replication, CRISPR guide identification and host transcriptional response. Using a proteostasis-focused knockout library, we identified shared and unique host factor requirements comparing four genetically distinct orthoflaviviruses. Transcriptional profiling revealed cell-intrinsic activation of the unfolded protein response as a universal feature of orthoflavivirus infection. In contrast, interferon stimulated gene activation occurred in paracrine fashion.

## Introduction

Orthoflaviviruses are a genetically diverse genus of viruses, which pose a significant burden to global public health infecting more than 400 million people annually [1,2]. Dengue viruses (DENV) account for the majority of infections. The orthoflavivirus genus includes a large group of tick-borne orthoflaviviruses, which are prevalent in Europe and Asia, and a genetically distinct, large group of mosquito-borne orthoflaviviruses, which are prevalent in tropical regions in Asia, Africa and South America [2–5]. Geographic distribution of these viruses will likely expand with climate change [6]. Primary hosts for mosquito-borne orthoflaviviruses range from humans and non-human primates (DENV, yellow fever virus (YFV), and Zika virus (ZIKV)), to birds (West Nile virus (WNV) and Japanese encephalitis virus). In addition to these variations in transmission cycle mediated by distinct insect vectors and mammalian hosts, orthoflaviviruses also vary in their human pathology. Severe cases of WNV can cause neurotropic pathologies such as encephalitis, ZIKV infection can cause congenital disorders including microcephaly, and severe DENV and YFV infections can result in hemorrhagic fever.

Despite differences in their transmission cycle and pathologies, orthoflavivirus replication strategies share commonalities. These positive-sense single-stranded RNA viruses are enveloped, with a genome approximately 11 kb in length [7]. The viral RNA, similar to messenger RNA, contains a 5′ cap, and encodes 10 viral proteins (3 structural proteins, 7 nonstructural proteins) translated as a single, large viral polyprotein. Orthoflaviviruses are among a small group of positive-sense RNA viruses that lack a poly-A tail. Cotranslational insertion of the polyprotein into the endoplasmic reticulum (ER) membrane and cleavage by host and viral proteases result in significant membrane reorganization to create invaginated replication organelles, which play a central role in viral genome amplification [8]. Given the complexity of the polyprotein structure, with at least 18 transmembrane domains, its biogenesis is highly dependent on cellular components.

High throughput CRISPR/Cas9, proteomic and siRNA screens have identified ER-resident host factors involved in proteostasis and quality control (QC) that are essential for orthoflavivirus infection by ensuring proper polyprotein biogenesis [9–17]. These host factors represent promising targets for host-directed antiviral therapeutics active against multiple orthoflaviviruses [18–20]. Given the genetic diversity within the orthoflaviviruses, it is unknown whether they have evolved common strategies to co-opt cellular pathways to promote their replication or whether there are differences between their host factor dependencies. Several of these host factors are transcriptionally upregulated during the unfolded protein response (UPR), an ER stress response which is activated during DENV infection [21–23]. This suggests that orthoflaviviruses both induce the UPR and co-opt these factors to promote their replication.

Here, we developed an experimental strategy to systematically compare host factor requirements among divergent orthoflaviviruses, and simultaneously capture the host transcriptomic response, providing a more comprehensive view of orthoflavivirus host factor requirements, as well as insight into the host response to each viral challenge. This single-cell approach, which we termed **Q**uantification of **I**nfection and **C**RISPR guide sequencing (QIC-seq), combines CRISPR/Cas9 knockout with virus-inclusive transcriptomics. Our results functionally validate a core set of host factors involved in ER-proteostasis and quantify their contributions to infection of four distinct orthoflaviviruses. We identify shared cellular pathways induced by all tested orthoflaviviruses, provide evidence of virus-specific host-immune response to infection, and further interrogate the combinatorics of host response to viral challenge in each perturbation. QIC-seq provides an experimental strategy to systematically compare this diverse family of medically important viruses and includes non-polyadenylated viruses in combinatorial single cell RNA sequencing strategies.

## Results

**Fig 1:**
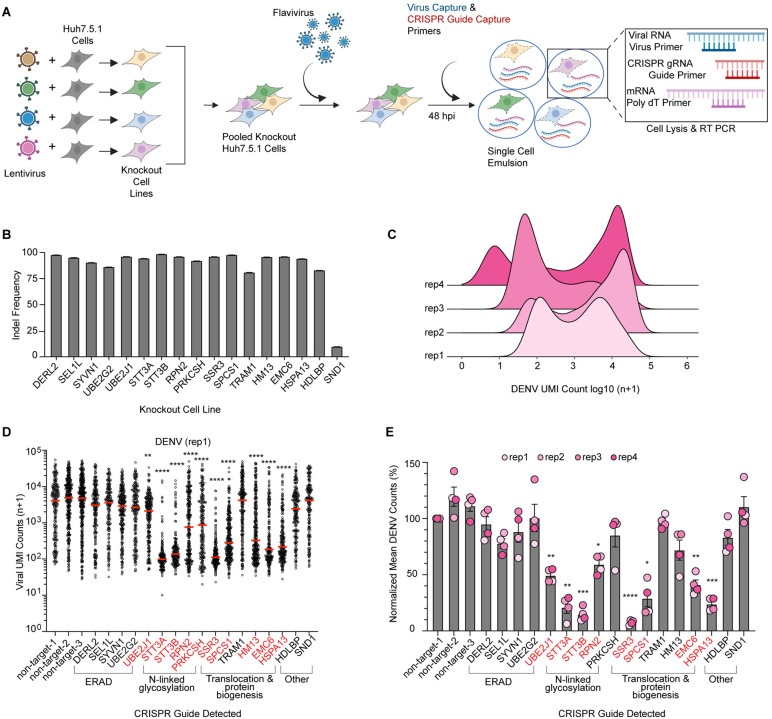
QIC-seq allows for intraviral comparison of host-factor requirements for DENV replication. **A,** Protocol design. Individual lentiCRISPRv2 plasmids were used to generate knockout cell lines that were pooled to generate a cell library. Cells were unchallenged or challenged with DENV (MOI 0.1) for 48 hours, harvested, and subject to 5′ capture using the 10X Genomics 5′ VDJ kit, with addition of primers targeting the 5′ region of the viral RNA, and the CRISPR guide scaffold. **B,** Indel frequency of Huh7.5.1 knockout cell lines. **C**, DENV UMI counts (log_10_ of n + 1) in all guide-detected, DENV challenged Huh7.5.1 cells, separated by each biological replicate of DENV QIC-seq screens. **D**, Viral counts plot of first biological replicate of DENV QIC-seq screens. Cells are plotted as circles by guide-detected and DENV UMI counts (log_10_ of n + 1). Red line represents the median value. Statistical significance was determined on log-transformed data using one-way ANOVA against non-target-1, correcting for multiple hypothesis testing using Dunnett’s test. Red text indicates perturbed genes in which viral replication is significantly reduced (p < 0.05). **** = p < 0.0001, ** = p < 0.01. **E,** Graph of the mean DENV count in guide-detected cells, divided by the mean DENV count in non-target-1 guide-detected cells for each biological replicate. Four biological replicates are graphed. Error bars represent standard error of the mean. Statistical significance determined using one-way ANOVA against non-target-1 correcting for multiple hypothesis testing using Dunnett’s test. Red text indicates perturbed genes in which viral replication is significantly reduced (p < 0.05). **** = p value <0.0001, *** = p value <0.001, ** = p value < 0.01, * = p value <0.05. Created in BioRender. Dupzyk, **A.** (2026) https://BioRender.com/wagfn2e.

For development of QIC-seq (Fig 1A) we chose to focus on cellular factors that, in prior genome-scale screens were identified as having a role in DENV infection, and, we hypothesized, might promote polyprotein biogenesis and insertion in the ER membrane based on cellular localization or function. These include proteins involved in ER-associated protein degradation (ERAD), N-linked glycosylation, translocation and protein biogenesis [11,13,14,24]. Knockout cell lines of each host gene were generated by transduction of lentiviruses encoding single guide RNAs (sgRNAs) into Huh7.5.1 cells. Human hepatoma Huh7.5 and derivate cell lines including Huh7.5.1 are defective in RIG-I signaling and are frequently used to study orthoflavivirus biology [25–27]. In total, 20 unique knockout cell lines were generated, each with a sgRNA targeting either a gene encoding a host factor previously identified as important in DENV infection, or a non-targeting (NT) guide as control (S1 Table). Amplicon sequencing of the targeted loci revealed efficient gene editing with insertion-deletion (indel) frequencies ranging between 80% and 95% for all sgRNAs except for the sgRNA targeting SND1, which showed less than 10% frequency (Fig 1B). Individual cell lines were counted and pooled in equal amounts. The resulting Huh7.5.1 cell library was then either mock infected, or infected with DENV type 2, for 48 hours at an MOI of 0.1.

Cells were collected and prepared for single-cell RNA sequencing using the 10X Genomics 5′ VDJ platform. To allow for the amplification of CRISPR guide RNA and DENV genomic RNA, we added capture primers annealing to the conserved loop in the sgRNA and to the 5′-end of the DENV genome, respectively [28]. The modification in the protocol ensures that, like the polyadenylated mRNA, sgRNAs and DENV genomic RNA cDNAs are captured and incorporate a unique modular identifier (UMI) and a cell barcode. This method enables simultaneous evaluation of viral RNA levels and identification of host perturbations, along with measurement of host cell gene expression (Fig 1A). After reverse transcription and cDNA amplification, cDNAs encoding the 5′ end of the viral genome and the sgRNA were size separated from cDNAs derived from host mRNA (transcriptome) before processing both libraries for single cell sequencing. Alignment of the reads against the human genome as well as custom gene references including the viral genomes generates a matrix of mRNA expression values for each cell, as well as a table with viral UMI counts and CRISPR guide identification for each cell, enabling quantification of the effects of CRISPR perturbation on viral replication, and insight into the host transcriptomic response.

### DENV replication is quantifiable in guide-detected cells

Following quality control thresholding and doublet elimination (cells with multiple distinct guides), we analyzed DENV counts in 10,828 DENV challenged Huh7.5.1 cells across 4 biological replicates. As a control, we similarly analyzed a cell population that was not infected by DENV (cells from unchallenged dish). As expected, in these 5,070 cells, there were no detectable DENV UMI counts in > 99% of cells (S2 Table). In contrast, DENV UMI counts were readily detectable in DENV challenged cells indicative of viral RNA replication (Fig 1C). To assess the potential contribution of ambient viral RNA to DENV UMI counts, we spiked in cells unchallenged with DENV just prior to droplet generation in one of the replicates (rep4) (S1A Fig). These cells could be identified by a distinct sgRNA not previously included in the initial pooled cell library. DENV reads in this population of 286 cells were low (ranging from 0-83 DENV counts, with a median value of 6) indicating that the large majority of reads in individual droplets originates from viral RNA actively produced by the cell itself rather than ambient viral RNA (S1B Fig).

Separating cells based on the identified sgRNA enables comparison of how individual host factor knockouts impact DENV viral replication (Fig 1D). Compared to non-target controls, most cells expressing sgRNAs against the catalytic subunits of the oligosaccharyltransferase (OST) complex, STT3A and STT3B, showed an approximately 100-fold reduction in DENV UMIs. This is in line with their previously established essential role for DENV RNA replication [11,14]. A similarly strong reduction in DENV replication was observed in cells with guides targeting SSR3, EMC6 and HSPA13, while targeting UBE2J1, RPN2, PRKCSH and SPCS1 resulted in more moderate reductions. The number of cells retrieved and the median DENV count in non-target guide detected cells varied between biological replicates, however, the significantly reduced DENV replication in cells with guides targeting STT3A, STT3B, SSR3, SPCS1, EMC6, and HSPA13 was observed in all replicates, highlighting the robustness of this screening method (Figs 1E, S1C–S1E). In addition, identical host-factor dependencies were observed between variable sequencing depths of the same viral and CRISPR library (DENV rep1, shallow) (S1F Fig, S3 Table). Thus, we have established QIC-seq as an experimental strategy that allows for quantitative intraviral comparisons of host factor requirements.

### Divergent orthoflaviviruses have dependencies on unique host factors

The choice of ER-proteostasis host factors in our library was based on prior work, which focused on DENV [11,14]. However, the conservation of these host factor requirements across orthoflaviviruses has not been systematically characterized. To close this knowledge gap, we applied QIC-seq to determine the effect of knockout of host factors on viral replication and host response. Our Huh7.5.1 cell library was challenged with Langat virus (LGTV), a naturally attenuated tick-borne encephalitis virus [29], WNV or YFV for 48 hours, and again subject to capture and QIC-seq library preparation. The multi-virus Huh7.5.1 QIC-seq screen contained 1,671, 2,171, and 3,602 LGTV, WNV, and YFV challenged cells, respectively, which we merged with the initial 11,114 DENV challenged and 5,070 unchallenged cells (S2 Table) to compare host transcriptional response and viral replication across viruses. Viral UMI counts were detectable in all LGTV, WNV and YFV challenged cells (S2 Table).

**Fig 2:**
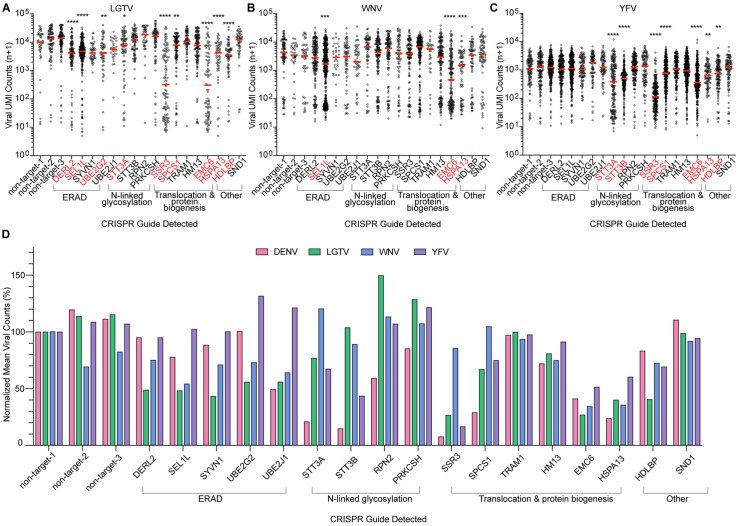
Divergent orthoflaviviruses have unique host factor dependencies. **A**, Viral counts plot of Huh7.5.1 cells challenged with LGTV at an MOI of 20 for 48 hrs. Cells, represented as circles, are plotted by guide-detected and LGTV UMI counts (log_10_ of n + 1). Red line represents median value. Statistical significance was determined on log-transformed data using one-way ANOVA against non-target-1, correcting for multiple hypothesis testing using Dunnett’s test. Red text indicates perturbed genes in which viral replication is significantly reduced (p < 0.05). **** = p value <0.0001, *** = p value <0.001, ** = p value < 0.01, * = p value <0.05. **B,** As in **A** except cells are challenged with WNV at an MOI of 0.1 for 48 hrs. **C**, As in **A** except cells are challenged with YFV at an MOI of 0.1 for 48 hrs. **D**, Mean viral UMI counts in guide-detected cells, normalized to the mean viral counts of non-target-1 guide-detected cells in each viral challenge.

Our analysis revealed commonalities and differences in host factor utilization by the tested orthoflaviviruses (Fig 2A-2D). Strikingly, while knockout of STT3A and STT3B results in strong decreases in YFV and especially DENV RNA replication, the other two orthoflaviviruses were less severely affected (Fig 2D). Conversely, knockout of ERAD components strongly inhibited LGTV and WNV RNA replication, had a moderate effect on DENV, and had a weaker effect on YFV replication (Fig 2A-2D). Knockout of the translocation related proteins SSR3, HSPA13 and EMC6, members of protein complexes required for insertion of transmembrane domains at the ER [30], led to strong decreases in viral replication for all orthoflaviviruses with the notable exception of SSR3 for WNV (Fig 2B, 2D).

### Universal upregulation of the unfolded protein response by orthoflaviviruses

QIC-seq combines CRISPR perturbation with a transcriptional readout of cellular mRNA during viral infection. For each viral challenge, cells were classified as highly, lowly or moderately infected based on viral UMI counts (see materials and methods), and differential gene expression analysis was performed between cells categorized as highly infected and lowly infected within the same viral challenge (S4–S5 Tables).

**Fig 3:**
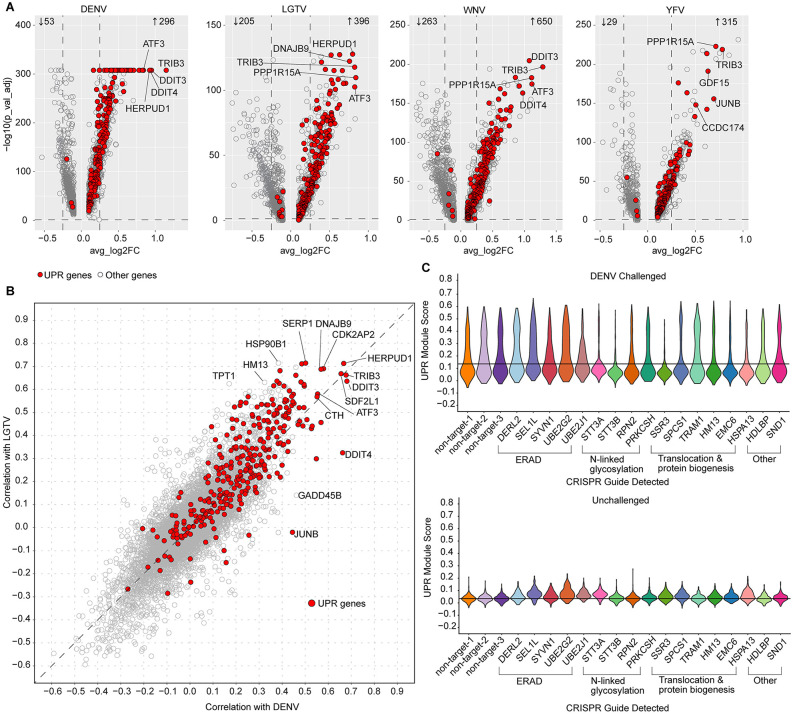
Orthoflaviviruses collectively induce the UPR in Huh7.5.1 cells. **A**, Volcano plots of differentially expressed genes in highly infected vs lowly infected Huh7.5.1 cells. UPR genes in red, all other genes in light grey. Number of downregulated genes are in top left corner; number of upregulated genes are in top right corner. Vertical dotted lines at ±0.25. Horizontal dotted line at -log_10_(0.05). DEGs identified using Seurat Findmarkers() function using default Wilcoxon Rank Sum test, with cutoffs set at log2fc ≥ ±0.25 and adjusted p value < 0.05. **B**, Genes plotted by Spearman’s correlation coefficient value with LGTV and DENV viral counts in Huh7.5.1 cells. UPR genes in red. Dotted line at y = x. **C**, Top: Violin plot of UPR module scores in DENV-challenged Huh7.5.1 cells, grouped by guide detected. Line represents median value of module scores in non-target-1 guide-detected cells. Bottom: as in top, however, cells are unchallenged.

Gene ontology on upregulated genes revealed the UPR and ERAD as the most enriched categories for upregulated genes in highly infected cells during all viral challenges (S6 Table). This is in line with prior reports indicating that UPR activation is a dominant transcriptional response during orthoflavivirus infection [31]. No obvious activation of interferon stimulated genes was observed in the Huh7.5.1 cells, as expected due to defective RIG-I signaling. Additional pathways that were significantly enriched in cells highly infected with DENV, LGTV and WNV included pathways corresponding to ER-to-Golgi trafficking, Golgi vesicle transport, and autophagosome assembly. To facilitate further comparative analysis, we assembled a list of genes that have been experimentally shown to be transcriptionally upregulated during ER stress (UPR module) [32,33] (S7 Table). UPR genes accounted for a large portion of upregulated genes in cells highly infected with DENV (38%) (Fig 3A). Similarly, 31%, 20%, and 19% of upregulated genes in cells highly infected with LGTV, WNV and YFV, respectively, corresponded to the UPR master list (Fig 3A).

To complement the differential gene expression analysis described above, we calculated correlation values between viral UMI counts and host mRNA counts. For DENV, the highest values were found in canonical UPR genes including TRIB3, DDIT3, DDIT4 and HERPUD1 (Fig 3B). Comparison of the correlation values between our study and a prior DENV single-cell study showed strong consistency (S2A Fig) [34]. To explore similarities and differences between orthoflaviviruses we plotted the correlation values of DENV counts with counts of the more distantly related LGTV(Fig 3B). Most genes grouped along the y = x line highlighting that DENV and LGTV induce a similar transcriptional response. Some exceptions such as GADD45B and JUNB correlation with DENV counts and TPT1 correlation with LGTV counts were noted.

As a quantitative measure of the gene set activity of the UPR pathway, we calculated module scores using the UPR list for each cell. Cells challenged with all viruses showed increases in UPR module score when compared to unchallenged cells (S2B Fig). To further analyze viral induction of the UPR, we assembled a list of genes shown to be upregulated during activation of specific UPR branches (ATF6, PERK and IRE1 modules) [32]. We observed an increase of all three branches with no readily apparent differences among the different viruses (S2C Fig). Correlation analysis showed that cells with high viral-UMI reads also displayed high UPR module scores suggesting that the upregulation is due to a cell-intrinsic mechanism and not paracrine signaling (S2D Fig).

We next categorized cells based on the detected sgRNA, which allowed us to determine the effect of gene knockout on UPR activation both in DENV-challenged and unchallenged samples (Fig 3C). In unchallenged cells, knockout of several genes including SEL1L, UBE2G2, and STT3A moderately increased the UPR module score compared to control, non-targeting sgRNAs (Fig 3C bottom panel). In line with this, differential gene expression analysis showed that the widely used UPR marker HSPA5 (also named BiP/GPR78) was upregulated upon knockout of these genes compared to non-targeting sgRNAs although overall few genes met the significance and fold-change thresholds (S5 Table). DENV-challenge strongly increased the UPR module score as was apparent in cells containing the non-targeting sgRNAs (Fig 3C upper panel). Cells containing sgRNAs targeting the host factors that most impacted DENV replication, including STT3A, STT3B and SSR3, displayed much reduced activation, indicating that DENV infection is the main driver of UPR activation. In agreement with this, cells containing sgRNAs targeting DENV-essential host factors showed a decreased expression in multiple UPR genes when compared to virally challenged non-targeting guide-detected cells (S5 Table).

Together, these data highlight that divergent orthoflaviviruses strongly induce the UPR and demonstrate the ability to assess the effect of CRISPR perturbation on the transcriptional response induced by viral infection.

### Optimized QIC-seq protocol for pooled library generation

**Fig 4:**
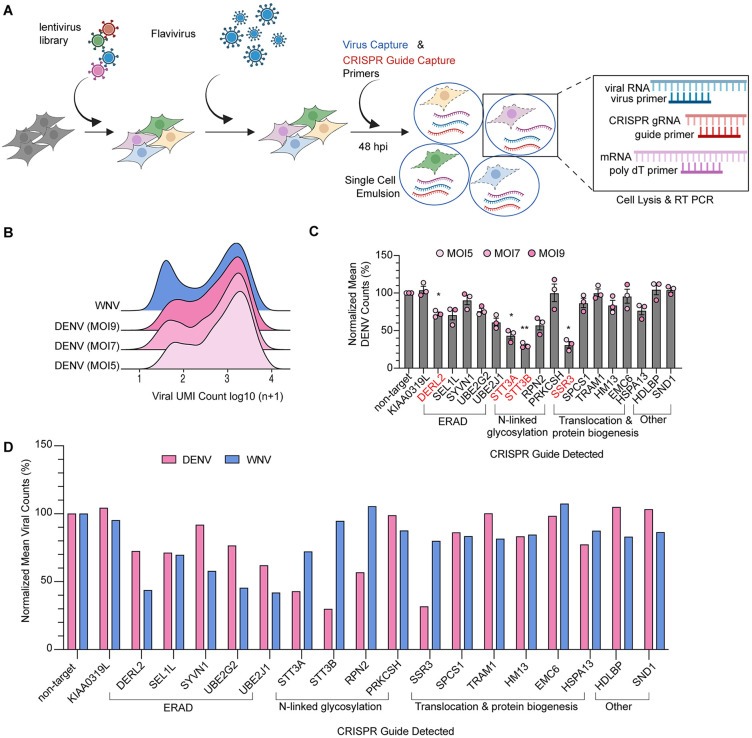
DENV replication is dependent on STT3A and STT3B in HAP1 cells, while WNV replication is dependent on ERAD machinery. **A**, Optimized QIC-seq protocol design. LentiCRISPRv2 plasmid library was used in single transduction to generate a cell library. Cells were unchallenged, or challenged with DENV or WNV for 48 hours, harvested, and subject to 5′ capture using the 10X Genomics 5′ VDJ kit, with addition of primers targeting the 5′ region of the viral RNA, and the CRISPR guide scaffold. **B**, UMI counts (log_10_ of n + 1) in viral challenged HAP1 cells, separated by virus, and infection. DENV-challenged cells are challenged at an MOI of 5, 7, and 9, respectively. Cells challenged with WNV at an MOI of 2.5. **C**, Mean DENV count in guide-detected cells, divided by mean DENV count in non-target guide-detected cells for each infection. Three infections are graphed with error bars representing standard error of the mean. Statistical significance determined using one-way ANOVA against non-target correcting for multiple hypothesis testing using Dunnett’s test. Red text indicates perturbed genes in which viral replication is significantly reduced (p < 0.05). ** = p value < 0.01, * = p value <0.05. **D**, Mean viral count in HAP1 guide-detected cells, divided by the mean viral count in non-target guide-detected cells for each infection. Three infections are included in the DENV challenge. Created in BioRender. Dupzyk, **A.** (2026) https://BioRender.com/g8itzp9.

Initial QIC-seq screens were performed by combining individually generated knockout cells, allowing us to determine gene editing efficiency of the individual sgRNAs and ensure each cell received a distinct sgRNA. To optimize this protocol for scalability and single-step cell library generation, we generated a single-vector lentiviral CRISPR library targeting the same 17 genes with 4 guides per gene, along with four non-targeting guides and four guides targeting the AAVR receptor (KIAA0319) as controls (S1 Table). This substantially quickens the cell library generation phase, streamlines the QIC-seq protocol and renders it compatible with larger CRISPR libraries (Fig 4A). To compare the transcriptional responses to orthoflavivirus infection and their dependencies on host factors between different cell types, we chose to introduce the library in HAP1 cells, which have been used in prior genetic screens for orthoflaviviruses and are IFN-signaling compentent [11,35]. HAP1 cells were either challenged with virus, or unchallenged for 48 hours, and subject to QIC-seq library preparation.

In total 2,972 unchallenged HAP1 cells were compared to 6,133 DENV challenged and 3,770 WNV challenged HAP1 cells. The three DENV infections were performed using slightly different MOIs of 5, 7, and 9, whereas the WNV infection was performed once at an MOI of 2.5. Similar to Huh7.5.1 infections, viral counts were detected in all challenged cells (Fig 4B, S8 Table). To analyze the phenotypic consequences of perturbation on orthoflavivirus replication in HAP1 cells, we plotted cells by viral counts and guide detected (S3A–S3B Fig). Generally, dependency profiles for DENV were similar to those observed in Huh7.5.1 cells with knockout of STT3A, STT3B, and SSR3 showing the most pronounced decrease in viral RNA counts and the components of the ERAD pathway showing a more moderate reduction. We observed consistent results between the three DENV infections (Figs 4C, S4C–S4E). WNV again showed strong dependence on ERAD components and no or only a slight dependence on STT3A and SSR3 (Figs 4D, S3B). Knockout of HSPA13 had a much more moderate effect on DENV replication in HAP1 cells, and EMC6 knockout did not affect RNA replication of either DENV or WNV in HAP1 cells while in Huh7.5.1 cells it resulted in strong replication defects (Fig 2D), suggesting differences in host factor dependencies involved in polyprotein biogenesis between cell types. Thus, we have optimized the QIC-seq experimental strategy and shown its utility in facilitating quantitative analysis of orthoflavivirus replication.

### QIC-seq reveals transcriptional differences in HAP1 cells challenged by DENV and WNV

We next investigated the transcriptional responses upon viral challenge in HAP1 cells. We classified cells by viral UMI counts in three categories as done previously (S9 Table) and identified genes differentially regulated between the highly and lowly-infected populations. Intriguingly, the transcriptional response in DENV-high cells compared to DENV-low cell resulted in just six genes, ASS1, SSR3, PSAT1, PCK2, SARS1, and ASNS, above a log2fc greater than 0.25 (S10 Table). Four of these genes (ASS1, PSAT1, SARS1 and ASNS) are involved in amino acid metabolism. In contrast, cells with high WNV counts showed 84 genes upregulated, and 83 genes downregulated according to the same criterion, despite comparable levels of infection between the two challenges (S4A Fig, S11 Table).

**Fig 5:**
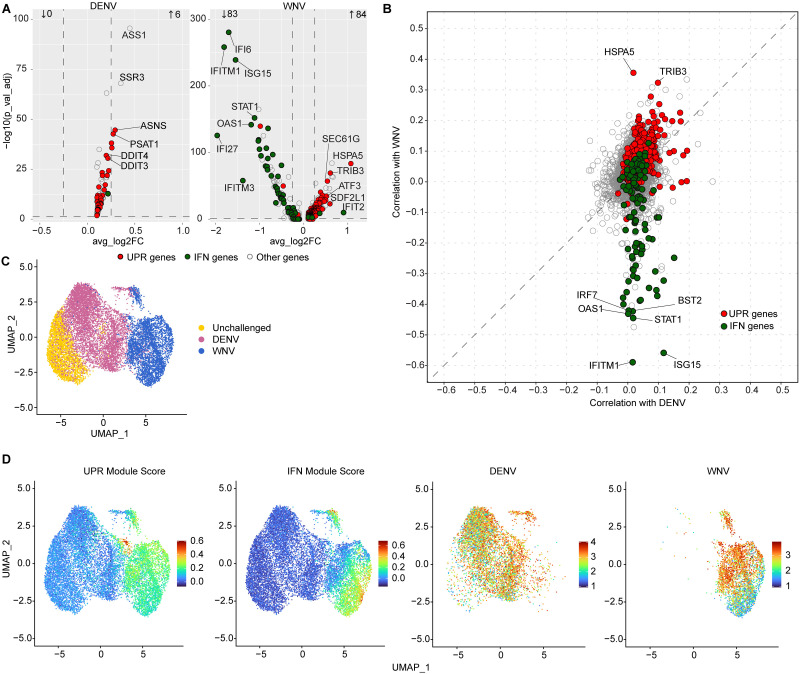
WNV challenge induces UPR and an IFN response in HAP1 cells. **A**, Volcano plots of differentially expressed genes in highly infected vs lowly infected HAP1 cells. UPR genes in red, IFN genes in dark green. All other genes in light grey. Number of downregulated genes in top left corner, number of upregulated genes in top right corner. Vertical dotted lines at ±0.25. Horizontal dotted line at -log_10_(0.05). DEGs identified using Seurat Findmarkers() function using default Wilcoxon Rank Sum test, with cutoffs set at log2fc ≥ ±0.25 and adjusted p value < 0.05. **B**, Genes plotted by correlation value with WNV and DENV viral counts in HAP1 cells. Spearman’s correlation coefficient used. Dotted line at y = x. **C**, Feature plot of all HAP1 cells, colored by challenge. **D**, Feature plots of HAP1 cells. Features include: UPR module score in all HAP1 cells, IFN module score of all HAP1 cells, DENV UMI counts (log_10_ n + 1) in DENV challenged HAP1 cells, and WNV UMI counts (log_10_ n + 1) in WNV challenged HAP1 cells.

Gene ontology analysis of upregulated genes in cells highly infected with WNV showed strong enrichment for gene sets involved in the response to ER stress (UPR), and strong depletion in gene sets involved in the IFN response (S12 Table). Therefore, we assembled a list of IFN stimulated genes (IFN module) analogous to the UPR module [36,37]. Highly infected WNV cells exhibited an upregulation of 34 genes from the UPR master list including the UPR marker HSPA5 (Fig 5A). Strikingly, 44 IFN stimulated genes were downregulated in cells with high WNV counts, including well-characterized interferon stimulated genes such as ISG15 and IFITM1 (Fig 5A, S10 Table). In line with this, correlation analysis showed positive correlation between WNV counts and UPR genes and negative correlation with IFN stimulated genes (Fig 5B).

To determine the effect of gene knockout we performed differential gene expression analysis between cells that express non-targeting sgRNAs and cells that express gene-targeting sgRNAs. In unchallenged cells, very few genes were differentially expressed (S10 Table). For some of the sgRNAs, we found downregulation of its target mRNA likely because indel formation can trigger nonsense mediated mRNA decay due to a premature termination codon generation [38]. In the DENV challenged sample, cells containing sgRNAs against STT3B or SSR3 displayed downregulation of ASS1 compared to non-target guides. This likely reflects the strong activity of STT3B and SSR3 sgRNAs in preventing DENV-replication thereby preventing ASS1 upregulation. In contrast, for WNV, none of the sgRNAs resulted in downregulation of UPR genes or upregulation of IFN genes. This could suggest that the genes targeted by sgRNA are less essential for WNV RNA replication. Alternatively, the low abundance of cells in the individual sgRNA subpopulations may have resulted in insufficient statistical power to detect differential gene expression.

Dimensional reduction analysis showed clear separation between unchallenged, DENV-challenged and WNV-challenged cells (Fig 5C). Module scores were again calculated for each cell, and elevated IFN and UPR module scores, especially through the PERK pathway, were observed in the WNV-challenged sample (Figs 5D, S4B–S4C). Plotting the WNV counts revealed a clear separation in the UMAP between highly infected cells and lowly-infected “bystander” cells (Fig 5D, right). Interestingly, the IFN module score was highest in the bystander cells suggesting that WNV infection triggers a paracrine interferon response that is suppressed by viral replication. IFNB1 was present in a small number of cells sequestered in the UMAP, while ISG15, IFI6, and IFITM1, the three most downregulated IFN stimulated genes in highly infected cells, followed a similar pattern as the IFN module score, in further support of paracrine IFN signaling (S4D Fig). Importantly, no increase in IFN module scores was observed in unchallenged, perturbed cells. (S4E Fig).

Within the DENV infected sample we observed little to no correlation between viral count numbers and the IFN or UPR module scores (S4F Fig). As observed for the Huh7.5.1 cells, WNV read counts were positively correlated with the UPR module score, however, the IFN module score was negatively correlated with WNV counts. Together, these data demonstrate that WNV infection triggers the UPR and IFN response in HAP1 cells, and that viral counts positively correlate with UPR activation and negatively with IFN response.

**Fig 6:**
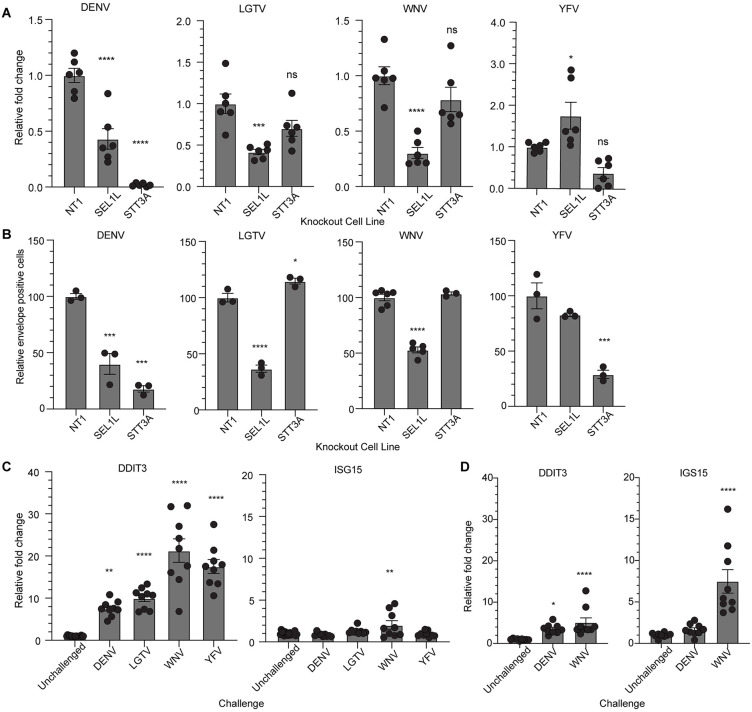
Orthogonal validation and confirmation of host factor dependencies and transcriptional responses. **A**, Relative viral RNA in Huh7.5.1 cells expressing non-target-1 (NT1), SEL1L, or STT3A gRNA as measured by virus-specific RT-qPCR. Cells were infected with DENV, LGTV, WNV or YFV at an MOI of 0.1, 20, 0.1 and 0.1, respectively for 48 hrs. Viral RNA was first normalized to a housekeeping gene (18S), then normalized to NT1. Statistical significance was determined using one-way ANOVA against NT1. Data are of 6 biological replicates. Each biological replicate was measured in technical duplicate. **B**, Relative infection in Huh7.5.1 cells expressing non-target-1 (NT1), SEL1L, or STT3A gRNA as measured by flow cytometry using virus Envelope antibodies. Cells were challenged with DENV, WNV or YFV at an MOI of 0.1, or LGTV at an MOI of 20 for 48 hrs. Percentage of infected cells (S11 Table) was normalized to NT1 and statistical significance was determined using one-way ANOVA against NT1. **C**, Relative expression of DDIT3 and ISG15 in Huh7.5.1 cells challenged with orthoflaviviruses as determined by RT-qPCR. Data representative of 9 biological replicates, performed in technical duplicate. Statistical significance determined using one-way ANOVA against uninfected sample. **D**, Relative expression of DDIT3 and ISG15 in HAP1 cells challenged with DENV at an MOI of 7 or WNV at an MOI of 2.5 for 48 hrs. Data representative of 9 biological replicates, performed in technical duplicate. Statistical significance was determined using one-way ANOVA against uninfected sample. **** = p value <0.0001, *** = p value <0.001, ** = p value < 0.01, * = p value <0.05.

To orthogonally validate the QIC-seq generated data, we challenged individual Huh7.5.1 cell lines from the original QIC-seq cell library (SEL1L knockout, STT3A knockout, and non-target-1) with all four orthoflaviviruses and measured relative viral replication by qPCR. In agreement with the viral counts plots (Figs 1D, 2A-2C), DENV and YFV replication was reduced in STT3A knockout cells, albeit only the reduction in DENV replication was statistically significant (Fig 6A). In contrast, LGTV and WNV replication was most reduced in SEL1L knockout cells. The effect of host gene knockout on viral infection was further assessed by immunostaining of infected cells using two orthoflavivirus Envelope (E) antibodies and quantification of infected cells using flow cytometry. In line with the qPCR experiments, STT3A knockout resulted in significant reductions of DENV and YFV but not LGTV and WNV infection. In contrast, SEL1L knockout resulted in significantly reduced DENV, LGTV and WNV but not YFV infection (Fig 6B, S11 Table).

To validate the observed host transcriptional responses induced by orthoflavivirus challenge, we challenged WT Huh7.5.1 and HAP1 cells again with all 4 viruses, and measured expression of DDIT3 and ISG15 by qPCR. In agreement with our QIC-seq data, all orthoflaviviruses induced the UPR in Huh7.5.1 cells, while little to no ISG15 upregulation was seen (Fig 6C). In contrast, WNV-challenged HAP1 cells showed a highly significant increase in DDIT3 and ISG15 expression, while DENV challenged HAP1 cells again showed little variation in either DDIT3 or ISG15 gene expression (Fig 6D).

## Discussion

We have developed a single-cell RNA sequencing strategy (QIC-seq) that allows for quantification of non-polyadenylated RNA virus replication and perturbation identification, while including single cell transcriptional analysis. Conventional CRISPR fitness screening approaches rely on bulk PCR amplification of sgRNA sequences from genomic DNA of cell populations to infer importance for viral fitness based on survival of viral challenge. In contrast, QIC-seq is an RNA-based approach that directly and sensitively measures the impact of host factor knockout on viral RNA accumulation and mRNA abundance. A recent study independently developed a similar approach to analyze SARS-CoV-2 replication and the interferon response in cells depleted of host factors identified as viral-binding proteins [39]. This approach has similarities with QIC-seq but is not suitable for non-polyadenylated viruses such as orthoflaviviruses, bunyaviruses and reoviruses.

RNA virus-inclusive single-cell RNA-sequencing has been instrumental in multiple studies monitoring heterogeneity in host response to viral challenge [39–43]. We have used QIC-seq to systematically compare host factor dependencies and transcriptional responses between divergent orthoflaviviruses and between distinct cell types. The inclusion of multiple viruses within one study using the same experimental conditions allows for meaningful inter-viral comparisons of host factor requirements and host responses. In addition, QIC-seq enables the monitoring of host transcriptional responses under viral challenge and amidst perturbation, including innate immune responses. Because host-directed antiviral development involves both direct targeting of host dependency factors, but also modulating immune responses, QIC-seq could provide a versatile tool to be used in this context [44].

Viral counts plots in this study revealed unique host factor requirements for orthoflaviviral replication. DENV RNA accumulation was consistently reduced in cells where STT3A or STT3B guides were detected. This strict DENV-dependence on the OST catalytic subunits is in line with prior reports [11,14]. The same observation was true of YFV replication. In contrast, WNV and LGTV showed little dependency on either STT3A or STT3B and instead depended more strongly on the core components of the ERAD machinery. This suggests that divergent orthoflaviviruses have evolved distinct mechanisms to utilize these host factors, although further studies are needed to reveal the molecular details.

Orthoflavivirus infection induces ER stress due to the high expression level and complex topology of the viral polyprotein. The strong positive correlation observed between viral UMI counts and the UPR module score in virus-infected cells demonstrates QIC-seq’s ability to identify cellular pathways induced during viral infection. The role of the UPR in promoting or antagonizing viral replication remains enigmatic [21]. While not undertaken here, QIC-seq is a useful tool that could be used to further our understanding of the UPR-orthoflavivirus relationship by generating and testing a CRISPR library specifically focused on components of the UPR pathway.

QIC-seq screens in HAP1 cells allowed us to investigate the IFN response to orthoflaviviruses, as these cells have intact RIG-I signaling, unlike Huh7.5.1 cells [25–27]. In WNV-challenged HAP1 cells, we found a strong activation of IFN stimulated genes suggesting that WNV infection results in the production of interferon. Intriguingly, we observed a negative correlation between WNV UMI counts and the IFN module score, likely indicative of paracrine IFN signaling, which is antagonized in highly infected cells, in agreement with a previous study [43]. This demonstrates the ability of virus-inclusive scRNA-seq to uncover complex relationships between viral infection and transcriptional responses, as bulk RNA-sequencing experiments would have masked the inverse relationship in WNV-infected cells. WNV nonstructural proteins have been shown to inhibit IFN signaling by preventing phosphorylation of STAT1 and STAT2 [45,46].

Interestingly, despite DENV UMI counts indicating productive infection in HAP1 cells, no correlation with the IFN module score was observed. This suggests that DENV infection did not result in production of IFN. This could be due to the superiority of DENV in preventing the initial step in the production of IFN, which is the detection of viral pathogen-associated molecular patterns (PAMPs) by pattern recognition receptors (PRRs) such as RIG-I and cGAS. Whether this reflects a passive mechanism by hiding PAMPs [47,48] or active suppression by inactivating PRRs [49] is unknown. Given the minimal UPR activation in DENV challenged HAP1 cells, it is also possible that lower efficiency of RNA replication compared to WNV infection contributed to the failure to trigger an IFN response, although the comparable infection levels makes this a less likely possibility.

In summary we present QIC-seq, a single-cell RNA-sequencing method in which orthoflavivirus replication is paired with perturbation identification and host transcriptomics. Using QIC-seq, we have compared divergent orthoflavivirus dependence on two major proteostasis-maintaining host complexes, the OST complex and ERAD machinery, across two cell lines and find unique host factor requirements for replication. We have identified the UPR as commonly upregulated during orthoflavivirus challenge, and observed a negative correlation between WNV infection and the host IFN response. This work furthers our knowledge of cellular requirements of a group of medically important viruses that cause significant burdens to global public health, with the potential to inform therapeutic design.

## Materials and methods

### Cell culture and viruses

Huh7.5.1 cells, a kind gift from F. Chisari, were cultured in DMEM media, 10% heat-inactivated fetal bovine serum (HI-FBS), and 1% penicillin and streptomycin (pen-strep). HAP1 cells, derived from near-haploid chronic myeloid leukemia cells, KBM7, were cultured in IMDM media, 10% HI-FBS, and 1% pen-strep. DENV2 (16681) was a generous gift from Dr. Karla Kirkegaard at Stanford University, and was adapted to HAP1 cells by serial passaging [11]. YFV (vaccine strain 17D) was generated by culturing Yellow Fever Vaccine YF-VAX 17D-204 vaccine. West Nile (Kunjin strain) was a generous gift from Dr. John F. Anderson at the Connecticut Agricultural Experiment Station. Langat Virus (TP12 strain) was a generous gift from Dr. Marshall Bloom at Rocky Mountain Laboratories.

### Generation of cell libraries using lentiviral transduction

Lentivirus was generated by co-transfecting the 𝚫VPR, VSV-G, and pAdVAntage lentivirus packaging plasmids along with CRISPR-guide containing lentiCRISPRv2 plasmids targeting genes of interest into 293FT cells using transIT-LT1 reagent (Mirus bio). Lentivirus was collected after 48 hours, and filtered using a 0.45-mm filter. The lentiCRISPRv2 plasmid was a gift from Feng Zhang (Addgene plasmid # 52961). All oligonucleotides for CRISPR sgRNA generation, QIC-seq, and indel frequency analysis were ordered from Integrated DNA Technologies (IDT).

To generate the Huh7.5.1 cell library, individual knockout cell lines were generated, combined, and used for the initial QIC-seq screen. CRISPR guide RNA sequences were selected from the human GeCKO library [50]. To generate the knockout cell lines, oligos (S1 Table) were annealed, phosphorylated, and ligated into BsmBI (New England Biosciences)-cleaved lentiCRISPRv2 before transforming into Stbl3 competent bacteria and used to generate lentivirus as above. Sequences were confirmed by Sanger sequencing. Huh7.5.1 cells were transduced with lentivirus plus 8 µg/ml protamine sulfate, and selected with 1 µg/ml puromycin after 48 hours. Cells were pooled at equal ratios. The SETD3 knockout cell line was generated as above, however was excluded from the cell library in order to represent a naïve knockout cell line in a DENV challenged environment.

To generate the HAP1 cell library, a lentivirus plasmid library was first generated, then used in a single transduction of HAP1 cells at a MOI of 0.3. To generate the HAP1 lentivirus plasmid library, 4 oligos encoding a sgRNA targeting each indicated gene and 4 non-targeting oligos were selected from the human Brunello library (S1 Table) [51], and purchased in a pooled format. A total of 1 ng of oligo pool was amplified using the forward primer: GGCTTTATATATCTTGTGGAAAGGACGAAACACC and the reverse primer: CTAGCCTTATTTTAACTTGCTATTTCTAGCTCTAAAAC at final concentrations of 500 nM and Q5 High Fidelity polymerase (New England Biolabs) according to manufacturer’s protocol with an annealing temperature of 60°C for 20 cycles. The resulting PCR product was gel extracted, and Gibson assembled into BsmBI-cleaved lentiCRISPRv2 at a 5:1 vector to insert ratio. Assembly reaction was transformed into Endura Duo electrocompetent cells, plated to calculate gene coverage, and a small number of colonies were confirmed by Sanger sequencing.

The plasmid library was used to generate lentivirus as above. Lentivirus was collected from transfected 293FT cell supernatant, centrifuged to remove cell debris, and concentrated using PEG-it (System Biosciences). HAP1 cells were transduced with concentrated lentivirus and 8 µg/ml protamine sulfate and selected using 1 µg/ml puromycin 48 hours after transduction.

### 10X Genomics kits

All Huh7.5.1 QIC-seq screens were conducted using the 10X Genomics Chromium Next GEM Single Cell 5′ Library and Gel Bead Kit (product code 1000167), the Chromium Single Cell 5′ Library Construction Kit (product code 1000020), the Chromium Next GEM Chip G single Cell Kit (product code 1000120), and the Single Index Kit T Set A (product code 1000213). All HAP1 QIC-seq screens were conducted using 10X genomics Chromium Next GEM Single Cell 5′ Kit v2 (product code 1000265), Chromium Next GEM Chip K Single Cell Kit (product code 1000287), Chromium 5′ CRISPR kit (product number 1000451), Library Construction Kit (product number 1000190), and the Dual Index Kit TT Set A (product number 1000215). Protocols followed were Chromium Next GEM Single Cell V(D)J Reagent Kits v1.1 User Guide Rev F, and Chromium Next GEM Single Cell 5′ v2 with Feature Barcode technology CRISPR Screening Rev B.

### Virus challenges and cell preparation for GEM generation

For Huh7.5.1 QIC-seq screens, 150,000 cells were seeded in 6 well plates in DMEM, 10% HI FBS, and 1% pen-strep, 16 hours prior to viral challenge. Cells were challenged for 48 hours with dengue virus type 2, yellow fever virus, West Nile virus, or Langat virus, all at an MOI of 0.1, except Langat which was used at an MOI of 20. After 48 hours, cells were collected and resuspended in single cell suspension at 1000 cells/µl in PBS and 0.04% BSA to prevent cell aggregation. Four biological replicates were completed for DENV challenge, and single replicates were completed for Langat, West Nile and yellow fever virus QIC-seq screens. Naïve SETD3 knockout cells were added into the cell suspension of a single DENV challenge biological replicate prior to GEM generation (replicate four). HAP1 QIC-seq screens were challenged as above, however dengue was used at an MOI of 5, 7, and 9, and West Nile virus was used at an MOI of 2.5. Recovery target from GEM generation was 10,000 cells.

### QIC-seq library preparation and sequencing

For Huh7.5.1 QIC-seq screens, primers targeting the CRISPR sgRNA scaffold and the 5′ end of the indicated viral genome were added into the cell + master mix suspension at a final concentration of 250nM before loading the chromium chip (see S13 Table for primers). cDNA synthesis, post GEM-RT clean up, and cDNA amplification all proceeded as written in the 10X protocol. In step 3.2 of Rev F, DNA select and SPRI clean up were modified to generate two separate libraries based on DNA size, using 0.6X SPRI beads. The gene expression library (GEX) contained DNA > 300 base pairs, and remained bound to beads. The viral/CRISPR library (V/C) contained DNA < 300 base pairs, and was collected from the supernatant. The GEX library followed the remaining 10X genomics protocol.

The V/C library was subject to READ2 placement PCR (see S13 Table for primers). Briefly, the SI PCR primer (10X genomics) and a primer containing the READ2 sequence was used to generate a DNA product that can be indexed for Illumina sequencing. After cDNA amplification (step 3 of rev F), and DNA selection and SPRI bead clean up (step 3.2 of rev F), 80µl of supernatant containing DNA < 300 bp in length was collected. To this, 70 µl of 2X SPRIselect (Beckman) was added for a 2.0X ratio. Beads were pelleted, washed with 80% ethanol, and resuspended in 45 µl of EB buffer (Qiagen).

For READ2 placement PCR, KAPA HiFi or KAPA HiFi Hotstart polymerase (Roche) was added to 10ng of DNA from the 45 µl of bead elution. Each reaction received the 10X SI_PCR primer, and the CRISPR guide READ2 placement primer as well as the respective virus READ2 placement primers at 245 nM each (see S13 Table for PCR conditions according to virus). Post PCR, 50 µl of 2X SPRIselect was added to the total 25 µl PCR volume for a 2.0X ratio. Beads were again pelleted, washed in 80% ethanol and resuspended in 40 µl EB buffer. From this, 5 µl were added into the indexing PCR reaction according to step 6 of rev F (see S13 Table for indexing PCR conditions).

HAP1 QIC-seq screen modification and sequencing was completed as above, however, no primer targeting the CRISPR sgRNA scaffold was added into the cell + master mix suspension as the Chromium 5′ CRISPR kit became commercially available. All V/C and GEX libraries were prepared and indexed separately before Illumina sequencing by Novogene with an intended read target of 25 million reads per V/C library, and 50 million reads per GEX library.

### Processing of Fastq files, sgRNA indexing library (feature reference), selection of cells, and Seurat Object (SO) processing

All fastq files generated from Illumina sequencing were analyzed using 10X Genomics Cell Ranger version 4. The transcriptomic library (GEX) and viral/CRISPR library (V/C) were designated Gene Expression and CRISPR Guide Capture, respectively, in all library files. A 20 base pair sequence of the 5′ end of the viral genome, in addition to CRISPR guide sequences were included in the Feature references file. Briefly, a feature barcode sequence (BC) targeting the guide RNA scaffold (GTTTTAGAGCTAGAA) was provided, along with variable sequences specific to each guide (see S14 Table for feature reference files). For each virus, 5′ genomic sequences were supplied as a feature, allowing for quantification from Cell Ranger’s CRISPR analysis output. Gene Expression fastq files were aligned to the Homo sapiens.GRCh38.99 genome. A final list of cells with a single type of CRISPR guide detected, meeting cell quality control standards was generated as follows and used in the viral counts plots and Seurat data analysis: first, Cell Ranger generated Protospacer Calls Per Cell file was used to identify a list of cells with a distinct CRISPR guide called. Next, cells present in the Filter Feature Barcode Matrix corresponding to the list of cells above is generated, and further subset according to cell quality control standards (S15 Table, S5–S6 Figs). Virus counts were taken from the Filter Feature Barcode Matrix.

Data was analyzed using Seurat v.4.4.0. Seurat objects (SOs) were generated for each QIC-seq screen, and merged to generate a single SO for each cell line. For Huh7.5.1 QIC-seq screens, SOs were merged and split by type of viral challenge before undergoing SCTransformation (version 2) with cell cycle and mitochondrial mapping percentage regressed out. Lastly, integration was performed using Seurat IntegrateData() function with default parameters.

For HAP1 cells, all SOs were merged to a single SO, normalized, and variable features were identified using the vst selection method with 2000 features using default parameters. Data was scaled with cell cycle and mitochondrial mapping percentage regressed out. To cluster and visualize cells, Seurat RunPCA() function was used with default parameters. UMAPs were generated including the first 20 principal components as suggested by visual inspection of elbow plot, using default parameters.

### Master gene list generation

An unfolded protein response (UPR), and IFN stimulated gene (IFN) master gene list were generated and used to compare differentially expressed genes resulting from viral challenge (S7 Table). The UPR master list was generated from experimental results from Adamson et al. [32] and Reich et al. [33]. The IFN stimulated gene master list was generated from Shaw et al. [36] and Lumb et al. [37].

### QC feature scatter plots (violin plots)

All cells with a single guide detected are plotted for each QIC-seq screen. Cells with values outside indicated ranges as seen in S15 Table are removed from analysis.

### Viral UMI count distributions

Viral UMI counts were plotted as log_10_(n + 1) values using Seurat RidgePlot() function. In the Huh7.5.1 DENV QIC-seq screen, guide detected cells were plotted, grouped by biological replicate. In the Huh7.5.1 multi-orthoflavivirus and HAP1 QIC-seq screens, cells are grouped by viral challenge.

### Viral counts cell plot and normalized viral counts

Cells were grouped by detected CRISPR guide, and viral UMI counts (n + 1) were plotted on log scale using GraphPad PRISM. Red line represents the median value. The QIC-seq plot for DENV challenged HAP1 cells represents three MOIs combined. To graph normalized DENV counts, cells were grouped by CRISPR guides detected, and mean viral counts were divided by mean viral counts in non-target-1 for Huh7.5.1 guide detected cells belonging to the same QIC-seq screen or combined non-target for HAP1 guide detected cells belonging to the same QIC-seq screen. Error bars represent standard error of the mean. All four biological replicates depicted in Huh7.5.1 cells, and all three infections depicted in HAP1 cells. To graph normalized viral counts in all viral challenges, cells are grouped according to viral challenge and guide detected. Mean viral UMI counts (n + 1) are then divided by mean viral UMI counts of non-target-1-guide detected Huh7.5.1 cells or of combined non-target HAP1 cells belonging to the corresponding QIC-seq screen.

### Module scores

Module scores were assigned to each cell in the original processed Seurat Object based on the UPR, AFT6, PERK, IRE1, and IFN stimulated master gene list (S7 Table) using the Seurat function AddModuleScore(). All module scores were assigned prior to any subsetting and further analysis. The Seurat VlnPlot() function was used to plot the module score according to specific parameters, such as by viral challenge, or by guide.

### Differentially expressed genes and GO analysis

Genes were identified as differentially expressed using the Seurat FindMarkers() function, with a log2fc threshold of 0.1, using the default Wilcoxon Rank Sum test. Non-mitochondrial genes were then further subset using a cut off of log2fc ≥ ±0.25, and adjusted p value ≥ 0.05. The resulting differentially expressed genes were then used for GO analysis using the enrichGO() function from clusteprofiler [52].

### Indel frequency

Genomic DNA from approximately 8 million cells from each Huh7.5.1 knockout cell line was extracted using the Qiagen DNeasy Blood and Tissue kit. DNA(145 ng) of all but three knockout cell lines was subject to PCR in which a 200–280 base pair length of genomic DNA encoding the target site was amplified (see S16 Table for primers). In the case of the DERL2, SND1, and TRAM1 knockout cell lines, DNA was subject to PCR in which a 400–600 base pair length of genomic DNA encoding the target site was amplified. Genomic DNA encoding the sgRNA target site was amplified using the Q5 High Fidelity polymerase, and underwent cycling conditions according to manufacturer’s protocol, with an anneal temperature of 65°C for 40 cycles. Samples were sequenced by the Massachusetts General Hospital Center for Computational and Integrative Biology for CRISPR or complete amplicon sequencing. CRISPR sequencing results were analyzed using publicly available CRISPResso2 [53]. DERL2, SND1, and TRAM1 sequencing was returned as consensus sequences, and manually analyzed for likely loss-of-function.

### Dimensional reduction analysis and feature visualization

All feature plots were generated using Seurat’s FeaturePlot() function. The RNA assay was used to visualize viral counts, module scores, or indicated gene expression in cells.

### Feature scatter plots and correlations

All data sets were subset by viral challenge, and Spearman’s correlation coefficient was calculated between viral UMI counts and module scores in R. Cells were plotted using Seurat’s FeatureScatter() function. Viral UMI and gene expression correlation values found in this study and in Zanini et al. [34], were compared using Spearman’s correlation coefficient after first ensuring identical gene lists.

### Cell Binning based on viral counts

Cells corresponding to each viral challenge were binned by dividing the viral counts range into tertiles, and assigning equal number of cells to each tertile (high, medium, low). See S4 and S9 Tables for values. All DENV challenged cells from the same cell line were then combined and analyzed.

### Comparison with previous viscRNA-Seq data

Count matrices for both host and virus from published viscRNA-Seq experiments on DENV and ZIKV were obtained (F.Z. was leading author on that report, data also on GEO: GSE110496) [30]. Raw read counts from both host and virus were added to a grand total used as denominator for CPM normalization using scanpy [54]. Correlation of host gene expression with viral normalized read counts was performed using scipy [55] in line with the original analyses.

### Flow cytometry

Approximately 300,000 Huh7.5.1 non-target-1 or knockout cells were plated in 6 well dishes, and challenged with an MOI of 0.1 for DENV, WNV, and YFV-challenged cells, or an MOI of 20 for LGVT-challenged cells for 48 hours. For HAP1 infection percentages, 500,000 HAP1 cells were plated in 6 well dishes and challenged with DENV or WNV at an MOI of 7 or 2.5, respectively for 48 hours. Cells were fixed in 8% PFA in PBS for 15 min, and pelleted by centrifugation. Cells were permeabilized and washed using BD Bioscience’s Cytoperm (Cat. No. 554714). To detect orthoflavivirus envelope protein, cells challenged with DENV, WNV, or YFV were incubated with monoclonal anti-orthoflavivirus group antigen, clone D1-4G2-4–15 (BEI resources, NR-50327) at a ratio of 1:250 for 1 hr at 4C. To detect orthoflavivirus envelope protein in LGTV challenge cells, cells were incubated with monoclonal anti-Langat virus envelope glycoprotein, clone 5G5 (BEI resources, NR-40318) at a ratio of 1:250 for 1 hr at 4C. Cells were washed, and incubated with fluorescent secondary antibody for 30 min at 4C before resuspension in 2% BSA prior to flow cytometry. Analysis was completed using FlowJo software, see S11 Table for infection percentages. Each experiment consists of at least three biological replicates, and statistical significance was determined using one-way ANOVA.

### qPCR

To measure viral replication or host mRNA, 20,000 cells from WT cells, or indicated non-target-1 or knockout cell lines were plated in 96 well plates and challenged for 48 hours with an MOI of 0.1 for DENV, WNV, and YFV-challenged cells, and an MOI of 20 for LGTV-challenged cells. After 48 hours, cells were lysed, and viral RNA was reverse transcribed and amplified using the VAZYME Cells-to-CT, 2 step SYBR green kit (CL122–02) using manufacturer’s protocol and primers listed in S17 Table. Each experiment consists of at least three biological replicates. Values were graphed and statistical significance was determined using one-way ANOVA.

## Supporting information

S1 Table Oligonucleotide sequences used to generate cell libraries. (XLSX)

S2 Table Viral counts per cell for all Huh7.5.1 QIC-seq screens. (XLSX)

S3 Table Sequencing saturation values. (XLSX)

S4 Table Viral reads used to categorize Huh7.5.1 cells by infection level, and final cell counts for each category. (XLSX)

S5 Table Differentially expressed genes in Huh7.5.1 cells. (XLSX)

S6 Table GO analysis of differentially expressed genes in Huh7.5.1 cells. (XLSX)

S7 Table Gene modules. (XLSX)

S8 Table Viral counts per cell for all HAP1 QIC-seq screens. (XLSX)

S9 Table Viral reads used to categorize HAP1 cells by infection level, and final cell counts for each category. (XLSX)

S10 Table Differentially expressed genes in HAP1 cells. (XLSX)

S11 Table Infection percentages determined by quantification of orthoflavivirus Envelope protein using flow cytometry. (XLSX)

S12 Table GO analysis of differentially expressed genes in HAP1 cells. (XLSX)

S13 Table Primers and conditions used for 10X PCR. (XLSX)

S14 Table Feature reference sequences used for 10X Cellranger. (XLSX)

S15 Table Quality Control (QC) values used for all QIC-seq screens. (XLSX)

S16 Table Indel primer sequences and target amplicon information used to determine indel frequencies. (XLSX)

S17 Table Primer sequences used in orthogonal validation. (XLSX)

S1 Fig Replicate QIC-seq screens quantify the effect of knockout of select host genes on DENV RNA replication. **A**, Protocol design for spike in experiment. As in Fig 1**A**, however, naïve knockout Huh7.5.1 cells are added to the challenged cell library just before single cell emulsion. These “Spike In” cells are identified by the expression of a unique sgRNA. **B**, Ridgeplot of spike in experiment showing the distribution of DENV UMI counts (log_10_ of n + 1). **C-E**, Viral counts plots of remaining three biological replicates of DENV QIC-seq screen. Cells, represented as circles, are plotted by guide detected and DENV UMI counts (log_10_ of n + 1). Red line represents median value. Statistical significance was determined on log-transformed data using one-way ANOVA against non-target-1, correcting for multiple hypothesis testing using Dunnett’s test. **** = p value <0.0001, *** = p value <0.001, ** = p value < 0.01, * = p value <0.05. Red text indicates perturbed genes in which viral replication is significantly reduced (p < 0.05). **F**, First biological replicate of DENV QIC-seq screen (as in Fig 1D), at shallower sequencing depth. See S3 Table for sequencing depth information for each screen. Created in BioRender. Dupzyk, A. (2026) https://BioRender.com/p93p845.
(TIF)

S2 Fig Orthoflaviviruses induce the UPR in Huh7.5.1 cells. **A**, Genes plotted by correlation value with DENV in Zanini et al., 2018, and correlation value with DENV from this study. Values are Spearman’s correlation coefficients. Red circles denote genes corresponding to the UPR gene list, all other genes in grey. **B**, Violin plot of UPR module scores in Huh7.5.1 cells, split by challenge. **C**, Violin plot of module scores of UPR branches (ATF6, PERK, IRE1) in Huh7.5.1 cells, split by challenge. **D**, Feature scatter plots of Huh7.5.1 cells plotted by UPR module score and viral counts. Number in bottom right corner denotes Spearman’s correlation coefficient.
(TIF)

S3 Fig DENV and WNV viral counts plots. **A**, Cell plot of DENV challenged HAP1 cells. Circles represent cells. Cells plotted by guide-detected, and DENV UMI counts (log_10_ of n + 1). Red line represents median value. Cells challenged with DENV at an MOI of 5, 7, and 9 for 48 hrs were combined. Statistical significance was determined on log-transformed data using one-way ANOVA against non-target-1, correcting for multiple hypothesis testing using Dunnett’s test. **** = p value <0.0001, *** = p value <0.001, ** = p value < 0.01, * = p value <0.05. Red text indicates perturbed genes in which viral replication is significantly reduced (p < 0.05). **B**, As in **A**, however HAP1 cells are challenged with WNV at an MOI of 2.5 for 48 hrs. **C,** As in A, however only HAP1 cells challenged with DENV at an MOI of 5 are plotted. **D,** As in A, however only HAP1 cells challenged with DENV at an MOI of 7 are plotted. **E,** As in A, however only HAP1 cells challenged with DENV at an MOI of 9 are plotted.
(TIF)

S4 Fig WNV induces the UPR and in highly infected HAP1 cells and the IFN response in lowly infected HAP1 cells. **A**, Infection in HAP1 cells as measured by detection of orthoflavivirus Envelope protein using flow cytometry. Cells were challenged with DENV or WNV at an MOI of 7 and 2.5, respectively, for 48 hrs. Percentage of infected cells (S11 Table) graphed. **B**, Violin plot of UPR and IFN module scores in HAP1 cells, split by challenge. **C**, Violin plot of module scores of UPR branches (ATF6, PERK, IRE1) in HAP1 cells, split by challenge. **D**, Feature plots of HAP1 cells. Features include: IFNB1, ISG15, IFI6, and IFITM1 gene expression in all HAP1 cells. **E**, Violin plot of IFN module scores in unchallenged HAP1 cells, grouped by guide detected. Line represents median value of module scores in non-target guide-detected cells. **F**, Feature scatter plots of HAP1 cells plotted by UPR module score and viral counts. Number in top right corner denotes Spearman’s correlation coefficient.
(TIF)

S5 Fig QC: Cell quality control for Huh7.5.1 cell libraries used in QIC-seq. **A**-**H**, Violin plots for number of RNA features, counts of RNA, and percent mitochondrial genes. Cut offs were made according to each data set (see S15 Table for values used) before data sets were merged into final Seurat Object (SO). **A**, Unchallenged Huh7.5.1 cells. **B**, DENV-challenged Huh7.5.1 cells, biological replicate 1. **C,** DENV-challenged Huh7.5.1 cells, biological replicate 2. **D**, DENV-challenged Huh7.5.1 cells, biological replicate 3. **E**, DENV-challenged Huh7.5.1 cells, biological replicate 4. **F**, LGTV-challenged Huh7.5.1 cells. **G**, WNV-challenged Huh7.5.1 cells. **H**, YFV-challenged Huh7.5.1 cells.
(TIF)

S6 Fig QC: Cell quality control for HAP1 cell libraries used in QIC-seq. **A**-**E**, Violin plots for number of RNA features, counts of RNA, and percent mitochondrial genes. Cut offs were made according to each data set (S15 Table for values used) before data sets were merged into final Seurat Object (SO). **A**, DENV-challenged HAP1 cells (MOI 5). **B**, DENV-challenged HAP1 cells (MOI 7). **C**, DENV-challenged HAP1 cells (MOI 9). **D**, Unchallenged HAP1 cells. **E**, WNV-challenged HAP1 cells.
(TIF)

## References

[1] Bhatt S, Gething PW, Brady OJ, Messina JP, Farlow AW, Moyes CL, et al. The global distribution and burden of dengue. Nature. 2013;496(7446):504–7. doi: 10.1038/nature12060 23563266 PMC3651993

[2] Pierson TC, Diamond MS. The continued threat of emerging flaviviruses. Nat Microbiol. 2020;5(6):796–812. doi: 10.1038/s41564-020-0714-0 32367055 PMC7696730

[3] Dobler G, Gniel D, Petermann R, Pfeffer M. Epidemiology and distribution of tick-borne encephalitis. Wien Med Wochenschr. 2012;162(11–12):230–8. doi: 10.1007/s10354-012-0100-5 22699708

[4] Kwasnik M, Rola J, Rozek W. Tick-borne encephalitis-review of the current status. J Clin Med. 2023;12(20):6603. doi: 10.3390/jcm12206603 37892741 PMC10607749

[5] Hills SL, Poehling KA, Chen WH, Staples JE. Tick-borne encephalitis vaccine: recommendations of the advisory committee on immunization practices, United States, 2023. MMWR Recomm Rep. 2023;72(5):1–29. doi: 10.15585/mmwr.rr7205a1 37943707 PMC10651317

[6] Tabachnick WJ. Climate change and the arboviruses: lessons from the evolution of the dengue and Yellow Fever Viruses. Annu Rev Virol. 2016;3(1):125–45. doi: 10.1146/annurev-virology-110615-035630 27482902

[7] Barrows NJ, Campos RK, Liao K-C, Prasanth KR, Soto-Acosta R, Yeh S-C, et al. Biochemistry and molecular biology of flaviviruses. Chem Rev. 2018;118(8):4448–82. doi: 10.1021/acs.chemrev.7b00719 29652486 PMC5937540

[8] Paul D, Bartenschlager R. Flaviviridae replication organelles: oh, what a tangled web we weave. Annu Rev Virol. 2015;2(1):289–310. doi: 10.1146/annurev-virology-100114-055007 26958917

[9] Krishnan MN, Ng A, Sukumaran B, Gilfoy FD, Uchil PD, Sultana H, et al. RNA interference screen for human genes associated with West Nile virus infection. Nature. 2008;455(7210):242–5. doi: 10.1038/nature07207 18690214 PMC3136529

[10] Ma H, Dang Y, Wu Y, Jia G, Anaya E, Zhang J, et al. A CRISPR-based screen identifies genes essential for West-Nile-virus-induced cell death. Cell Rep. 2015;12(4):673–83. doi: 10.1016/j.celrep.2015.06.049 26190106 PMC4559080

[11] Marceau CD, Puschnik AS, Majzoub K, Ooi YS, Brewer SM, Fuchs G, et al. Genetic dissection of Flaviviridae host factors through genome-scale CRISPR screens. Nature. 2016;535(7610):159–63. doi: 10.1038/nature18631 27383987 PMC4964798

[12] Savidis G, McDougall WM, Meraner P, Perreira JM, Portmann JM, Trincucci G, et al. Identification of Zika virus and dengue virus dependency factors using functional genomics. Cell Rep. 2016;16(1):232–46. doi: 10.1016/j.celrep.2016.06.028 27342126

[13] Zhang R, Miner JJ, Gorman MJ, Rausch K, Ramage H, White JP, et al. A CRISPR screen defines a signal peptide processing pathway required by flaviviruses. Nature. 2016;535(7610):164–8. doi: 10.1038/nature18625 27383988 PMC4945490

[14] Lin DL, Cherepanova NA, Bozzacco L, MacDonald MR, Gilmore R, Tai AW. Dengue virus Hijacks a noncanonical oxidoreductase function of a cellular oligosaccharyltransferase complex. mBio. 2017;8(4):e00939–17. doi: 10.1128/mBio.00939-17 28720733 PMC5516256

[15] Shah PS, Link N, Jang GM, Sharp PP, Zhu T, Swaney DL, et al. Comparative flavivirus-host protein interaction mapping reveals mechanisms of dengue and zika virus pathogenesis. Cell. 2018;175(7):1931–1945.e18. doi: 10.1016/j.cell.2018.11.028 30550790 PMC6474419

[16] Lin DL, Inoue T, Chen Y-J, Chang A, Tsai B, Tai AW. The ER membrane protein complex promotes biogenesis of dengue and zika virus non-structural multi-pass transmembrane proteins to support infection. Cell Rep. 2019;27(6):1666-1674.e4. doi: 10.1016/j.celrep.2019.04.051 31067454 PMC6521869

[17] Ngo AM, Shurtleff MJ, Popova KD, Kulsuptrakul J, Weissman JS, Puschnik AS. The ER membrane protein complex is required to ensure correct topology and stable expression of flavivirus polyproteins. Elife. 2019;8:e48469. doi: 10.7554/eLife.48469 31516121 PMC6756788

[18] Heaton NS, et al. Targeting viral proteostasis limits influenza virus, HIV, and dengue virus infection. Immunity. 2016;44:46–58. 26789921 10.1016/j.immuni.2015.12.017 PMC4878455

[19] Qiao W, Xie X, Shi P-Y, Ooi YS, Carette JE. Druggable genome screens identify SPP as an antiviral host target for multiple flaviviruses. Proc Natl Acad Sci U S A. 2025;122(8):e2421573122. doi: 10.1073/pnas.2421573122 39969998 PMC11874179

[20] Verhaegen M, Croonenborghs M, Sorout N, Sartori A, Provinciael B, Meyen E, et al. Sec61 translocon inhibitor flavitransin blocks selectively dengue virus polyprotein insertion in the ER with pan-orthoflavivirus antiviral potency. Cell Rep. 2025;44(12):116642. doi: 10.1016/j.celrep.2025.116642 41348541

[21] Peña J, Harris E. Dengue virus modulates the unfolded protein response in a time-dependent manner. J Biol Chem. 2011;286(16):14226–36. doi: 10.1074/jbc.M111.222703 21385877 PMC3077624

[22] Lewy TG, Grabowski JM, Bloom ME. BiP: master regulator of the unfolded protein response and crucial factor in flavivirus biology. Yale J Biol Med. 2017;90(2):291–300. 28656015 PMC5482305

[23] Lewy TG, Offerdahl DK, Grabowski JM, Kellman E, Mlera L, Chiramel A, et al. PERK-mediated unfolded protein response signaling restricts replication of the Tick-Borne Flavivirus Langat Virus. Viruses. 2020;12(3):328. doi: 10.3390/v12030328 32197325 PMC7150897

[24] Ooi YS, Majzoub K, Flynn RA, Mata MA, Diep J, Li JK, et al. An RNA-centric dissection of host complexes controlling flavivirus infection. Nat Microbiol. 2019;4(12):2369–82. doi: 10.1038/s41564-019-0518-2 31384002 PMC6879806

[25] Blight KJ, McKeating JA, Rice CM. Highly permissive cell lines for subgenomic and genomic hepatitis C virus RNA replication. J Virol. 2002;76(24):13001–14. doi: 10.1128/jvi.76.24.13001-13014.2002 12438626 PMC136668

[26] Zhong J, Gastaminza P, Cheng G, Kapadia S, Kato T, Burton DR, et al. Robust hepatitis C virus infection in vitro. Proc Natl Acad Sci U S A. 2005;102(26):9294–9. doi: 10.1073/pnas.0503596102 15939869 PMC1166622

[27] Balsitis SJ, Coloma J, Castro G, Alava A, Flores D, McKerrow JH, et al. Tropism of dengue virus in mice and humans defined by viral nonstructural protein 3-specific immunostaining. Am J Trop Med Hyg. 2009;80(3):416–24. doi: 10.4269/ajtmh.2009.80.416 19270292

[28] Mimitou EP, Cheng A, Montalbano A, Hao S, Stoeckius M, Legut M, et al. Multiplexed detection of proteins, transcriptomes, clonotypes and CRISPR perturbations in single cells. Nat Methods. 2019;16(5):409–12. doi: 10.1038/s41592-019-0392-0 31011186 PMC6557128

[29] Rumyantsev AA, Murphy BR, Pletnev AG. A tick-borne Langat virus mutant that is temperature sensitive and host range restricted in neuroblastoma cells and lacks neuroinvasiveness for immunodeficient mice. J Virol. 2006;80(3):1427–39. doi: 10.1128/JVI.80.3.1427-1439.2006 16415020 PMC1346960

[30] Pleiner T, Tomaleri GP, Januszyk K, Inglis AJ, Hazu M, Voorhees RM. Structural basis for membrane insertion by the human ER membrane protein complex. Science. 2020;369(6502):433–6. doi: 10.1126/science.abb5008 32439656 PMC7547852

[31] Verhaegen M, Vermeire K. The endoplasmic reticulum (ER): a crucial cellular hub in flavivirus infection and potential target site for antiviral interventions. Npj Viruses. 2024;2(1):24. doi: 10.1038/s44298-024-00031-7 40295816 PMC11721386

[32] Adamson B, Norman TM, Jost M, Cho MY, Nuñez JK, Chen Y, et al. A multiplexed single-cell CRISPR screening platform enables systematic dissection of the unfolded protein response. Cell. 2016;167(7):1867–1882.e21. doi: 10.1016/j.cell.2016.11.048 27984733 PMC5315571

[33] Reich S, Nguyen CDL, Has C, Steltgens S, Soni H, Coman C, et al. A multi-omics analysis reveals the unfolded protein response regulon and stress-induced resistance to folate-based antimetabolites. Nat Commun. 2020;11(1):2936. doi: 10.1038/s41467-020-16747-y 32522993 PMC7287054

[34] Zanini F, Pu S-Y, Bekerman E, Einav S, Quake SR. Single-cell transcriptional dynamics of flavivirus infection. Elife. 2018;7:e32942. doi: 10.7554/eLife.32942 29451494 PMC5826272

[35] Labeau A, Simon-Loriere E, Hafirassou M-L, Bonnet-Madin L, Tessier S, Zamborlini A, et al. A genome-wide CRISPR-Cas9 screen identifies the dolichol-phosphate mannose synthase complex as a host dependency factor for dengue virus infection. J Virol. 2020;94(7):e01751–19. doi: 10.1128/JVI.01751-19 31915280 PMC7081898

[36] Shaw AE, Hughes J, Gu Q, Behdenna A, Singer JB, Dennis T, et al. Fundamental properties of the mammalian innate immune system revealed by multispecies comparison of type I interferon responses. PLoS Biol. 2017;15(12):e2004086. doi: 10.1371/journal.pbio.2004086 29253856 PMC5747502

[37] Lumb JH, Li Q, Popov LM, Ding S, Keith MT, Merrill BD, et al. DDX6 represses aberrant activation of interferon-stimulated genes. Cell Rep. 2017;20(4):819–31. doi: 10.1016/j.celrep.2017.06.085 28746868 PMC5551412

[38] Popp MW, Maquat LE. Leveraging rules of nonsense-mediated mRNA decay for genome engineering and personalized medicine. Cell. 2016;165:1319–22. 27259145 10.1016/j.cell.2016.05.053 PMC4924582

[39] Sunshine S, Puschnik AS, Replogle JM, Laurie MT, Liu J, Zha BS, et al. Systematic functional interrogation of SARS-CoV-2 host factors using Perturb-seq. Nat Commun. 2023;14(1):6245. doi: 10.1038/s41467-023-41788-4 37803001 PMC10558542

[40] Zanini F, Robinson ML, Croote D, Sahoo MK, Sanz AM, Ortiz-Lasso E, et al. Virus-inclusive single-cell RNA sequencing reveals the molecular signature of progression to severe dengue. Proc Natl Acad Sci U S A. 2018;115(52):E12363–9. doi: 10.1073/pnas.1813819115 30530648 PMC6310786

[41] Russell AB, Trapnell C, Bloom JD. Extreme heterogeneity of influenza virus infection in single cells. Elife. 2018;7:e32303. doi: 10.7554/eLife.32303 29451492 PMC5826275

[42] Russell AB, Elshina E, Kowalsky JR, Te Velthuis AJW, Bloom JD. Single-cell virus sequencing of influenza infections that trigger innate immunity. J Virol. 2019;93(14):e00500–19. doi: 10.1128/JVI.00500-19 31068418 PMC6600203

[43] O’Neal JT, Upadhyay AA, Wolabaugh A, Patel NB, Bosinger SE, Suthar MS. West Nile virus-inclusive single-cell RNA sequencing reveals heterogeneity in the Type I interferon response within single cells. J Virol. 2019;93(6):e01778-18. doi: 10.1128/JVI.01778-18 30626670 PMC6401468

[44] Kaufmann SHE, Dorhoi A, Hotchkiss RS, Bartenschlager R. Host-directed therapies for bacterial and viral infections. Nat Rev Drug Discov. 2018;17(1):35–56. doi: 10.1038/nrd.2017.162 28935918 PMC7097079

[45] Guo J-T, Hayashi J, Seeger C. West Nile virus inhibits the signal transduction pathway of alpha interferon. J Virol. 2005;79(3):1343–50. doi: 10.1128/JVI.79.3.1343-1350.2005 15650160 PMC544142

[46] Liu WJ, Wang XJ, Mokhonov VV, Shi P-Y, Randall R, Khromykh AA. Inhibition of interferon signaling by the New York 99 strain and Kunjin subtype of West Nile virus involves blockage of STAT1 and STAT2 activation by nonstructural proteins. J Virol. 2005;79(3):1934–42. doi: 10.1128/JVI.79.3.1934-1942.2005 15650219 PMC544092

[47] Espada-Murao LA, Morita K. Delayed cytosolic exposure of Japanese encephalitis virus double-stranded RNA impedes interferon activation and enhances viral dissemination in porcine cells. J Virol. 2011;85(13):6736–49. doi: 10.1128/JVI.00233-11 21525349 PMC3126492

[48] Uchida L, Espada-Murao LA, Takamatsu Y, Okamoto K, Hayasaka D, Yu F, et al. The dengue virus conceals double-stranded RNA in the intracellular membrane to escape from an interferon response. Sci Rep. 2014;4:7395. doi: 10.1038/srep07395 25491663 PMC4261170

[49] Aguirre S, Luthra P, Sanchez-Aparicio MT, Maestre AM, Patel J, Lamothe F, et al. Dengue virus NS2B protein targets cGAS for degradation and prevents mitochondrial DNA sensing during infection. Nat Microbiol. 2017;2:17037. doi: 10.1038/nmicrobiol.2017.37 28346446 PMC7457382

[50] Shalem O, Sanjana NE, Hartenian E, Shi X, Scott DA, Mikkelson T, et al. Genome-scale CRISPR-Cas9 knockout screening in human cells. Science. 2014;343(6166):84–7. doi: 10.1126/science.1247005 24336571 PMC4089965

[51] Doench JG, Fusi N, Sullender M, Hegde M, Vaimberg EW, Donovan KF, et al. Optimized sgRNA design to maximize activity and minimize off-target effects of CRISPR-Cas9. Nat Biotechnol. 2016;34(2):184–91. doi: 10.1038/nbt.3437 26780180 PMC4744125

[52] Yu G. Thirteen years of clusterProfiler. Innovation (Camb). 2024;5(6):100722. doi: 10.1016/j.xinn.2024.100722 39529960 PMC11551487

[53] Clement K, Rees H, Canver MC, Gehrke JM, Farouni R, Hsu JY, et al. CRISPResso2 provides accurate and rapid genome editing sequence analysis. Nat Biotechnol. 2019;37(3):224–6. doi: 10.1038/s41587-019-0032-3 30809026 PMC6533916

[54] Wolf FA, et al. SCANPY: large-scale single-cell gene expression data analysis. Genome Biol. 2018;19(15). 10.1186/s13059-017-1382-0 PMC5802054 29409532

[55] Virtanen P, Gommers R, Oliphant TE, Haberland M, Reddy T, Cournapeau D, et al. SciPy 1.0: fundamental algorithms for scientific computing in Python. Nat Methods. 2020;17(3):261–72. doi: 10.1038/s41592-019-0686-2 32015543 PMC7056644

