# Supplementary figures and images for: Dissecting the host determinants of orthoflavivirus infection using QIC-seq
Source: PLoS Pathog. 2026 Jun 26;22(6):e1014279. doi: 10.1371/journal.ppat.1014279 (PMC13327515; doi:10.1371/journal.ppat.1014279)

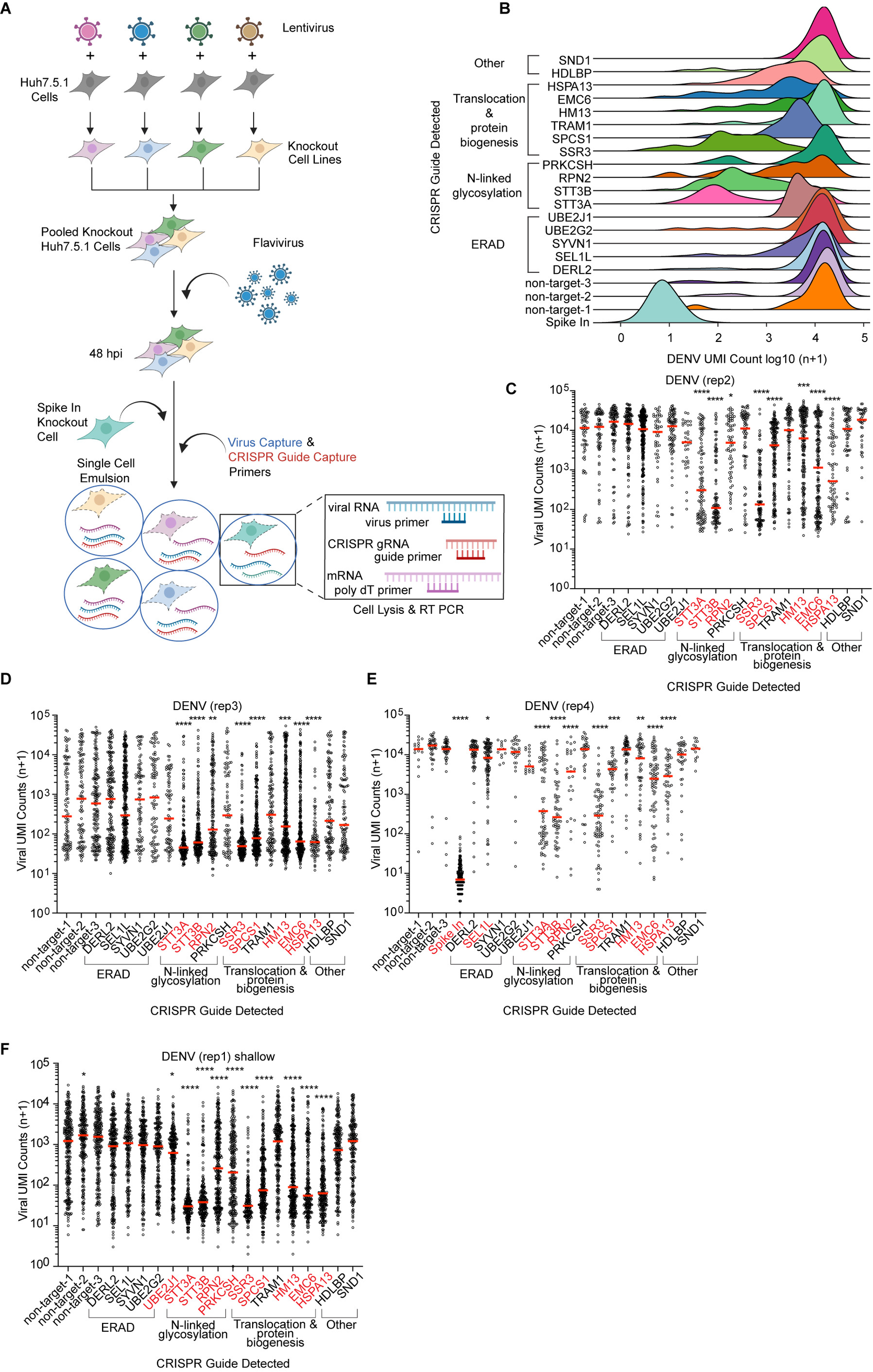

Supplement: S1 Fig — A, Protocol design for spike in experiment. As in Fig 1A, however, naïve knockout Huh7.5.1 cells are added to the challenged cell library just before single cell emulsion. These “Spike In” cells are identified by the expression of a unique sgRNA. B, Ridgeplot of spike in experiment showing the distribution of DENV UMI counts (log10 of n + 1). C-E, Viral counts plots of remaining three biological replicates of DENV QIC-seq screen. Cells, represented as circles, are plotted by guide detected and DENV UMI counts (log10 of n + 1). Red line represents median value. Statistical significance was determined on log-transformed data using one-way ANOVA against non-target-1, correcting for multiple hypothesis testing using Dunnett’s test. **** = p value <0.0001, *** = p value <0.001, ** = p value < 0.01, * = p value <0.05. Red text indicates perturbed genes in which viral replication is significantly reduced (p < 0.05). F, First biological replicate of DENV QIC-seq screen (as in Fig 1D), at shallower sequencing depth. See S3 Table for sequencing depth information for each screen. Created in BioRender. Dupzyk, A. (2026) https://BioRender.com/p93p845. (TIF) [file ppat.1014279.s018.tif]

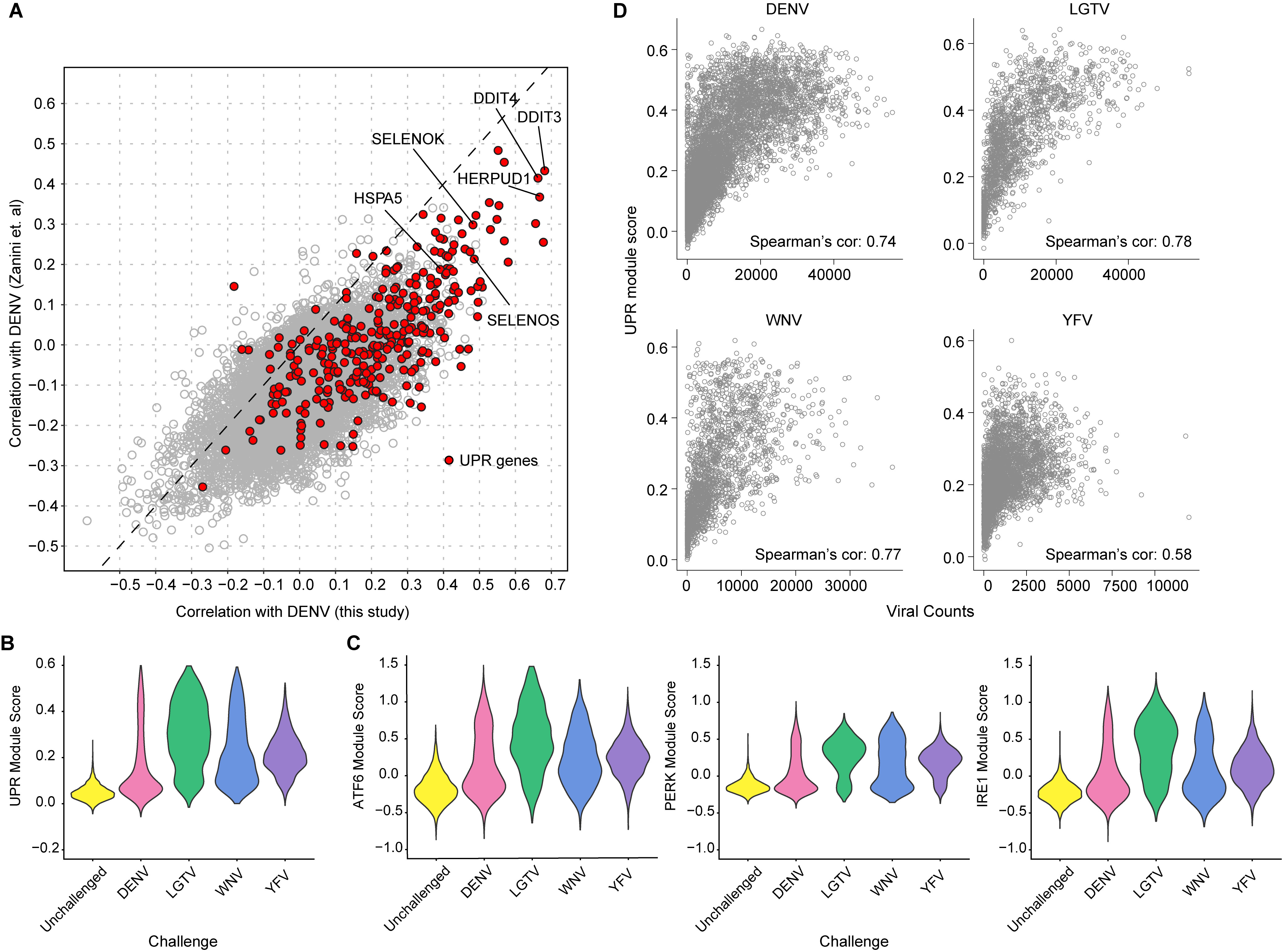

Supplement: S2 Fig — A, Genes plotted by correlation value with DENV in Zanini et al., 2018, and correlation value with DENV from this study. Values are Spearman’s correlation coefficients. Red circles denote genes corresponding to the UPR gene list, all other genes in grey. B, Violin plot of UPR module scores in Huh7.5.1 cells, split by challenge. C, Violin plot of module scores of UPR branches (ATF6, PERK, IRE1) in Huh7.5.1 cells, split by challenge. D, Feature scatter plots of Huh7.5.1 cells plotted by UPR module score and viral counts. Number in bottom right corner denotes Spearman’s correlation coefficient. (TIF) [file ppat.1014279.s019.tif]

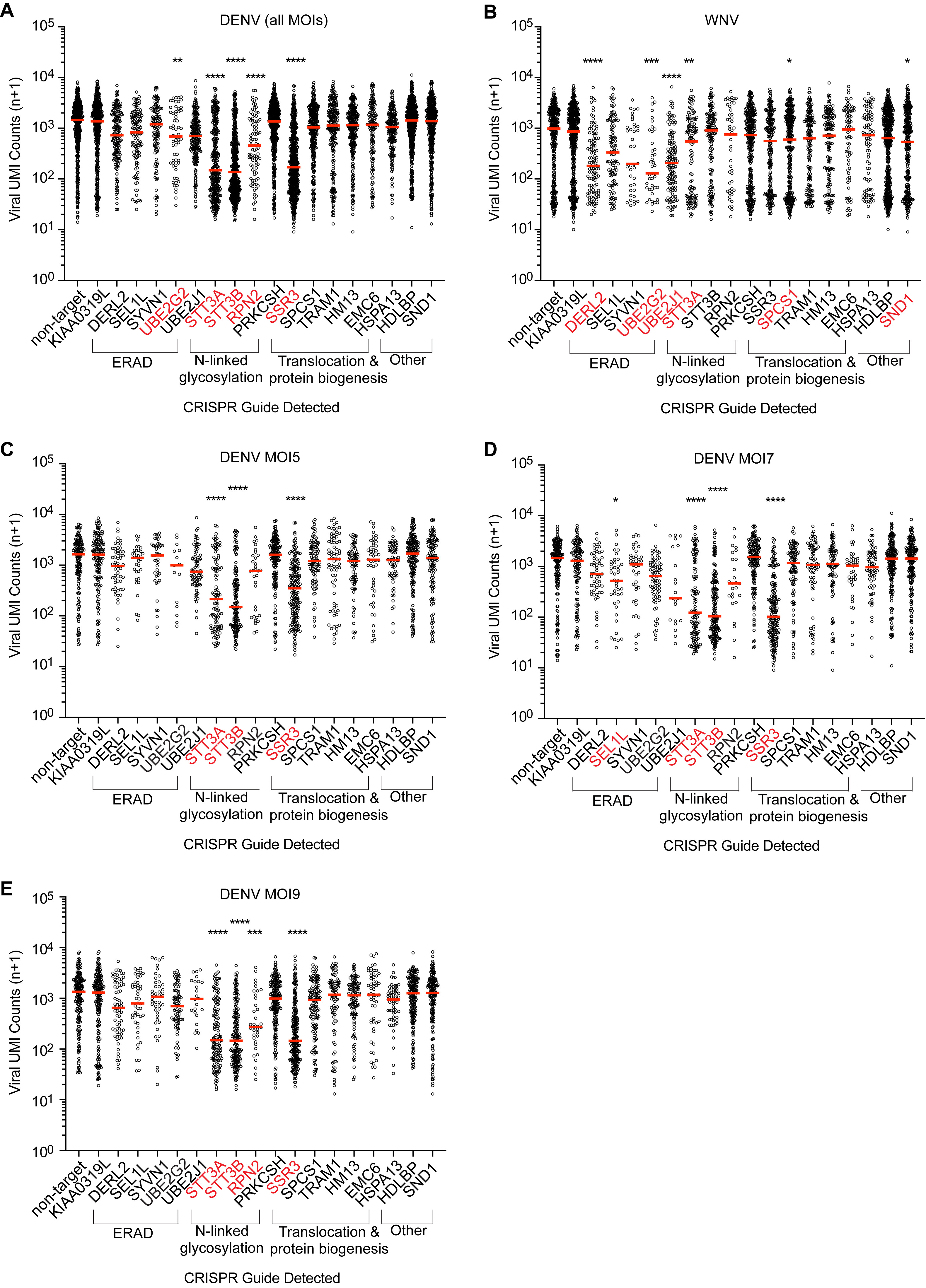

Supplement: S3 Fig — A, Cell plot of DENV challenged HAP1 cells. Circles represent cells. Cells plotted by guide-detected, and DENV UMI counts (log10 of n + 1). Red line represents median value. Cells challenged with DENV at an MOI of 5, 7, and 9 for 48 hrs were combined. Statistical significance was determined on log-transformed data using one-way ANOVA against non-target-1, correcting for multiple hypothesis testing using Dunnett’s test. **** = p value <0.0001, *** = p value <0.001, ** = p value < 0.01, * = p value <0.05. Red text indicates perturbed genes in which viral replication is significantly reduced (p < 0.05). B, As in A, however HAP1 cells are challenged with WNV at an MOI of 2.5 for 48 hrs. C, As in A, however only HAP1 cells challenged with DENV at an MOI of 5 are plotted. D, As in A, however only HAP1 cells challenged with DENV at an MOI of 7 are plotted. E, As in A, however only HAP1 cells challenged with DENV at an MOI of 9 are plotted. (TIF) [file ppat.1014279.s020.tif]

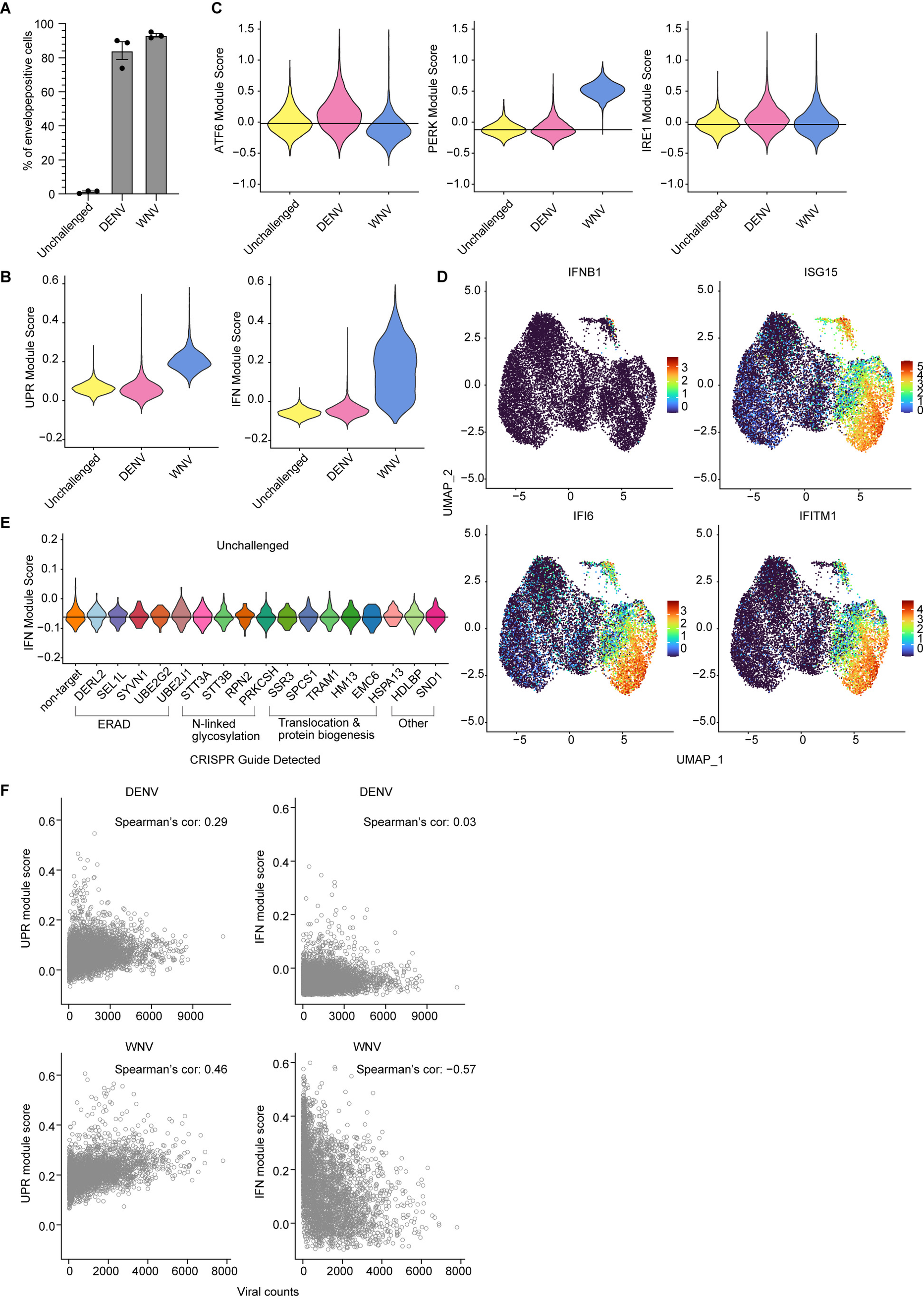

Supplement: S4 Fig — A, Infection in HAP1 cells as measured by detection of orthoflavivirus Envelope protein using flow cytometry. Cells were challenged with DENV or WNV at an MOI of 7 and 2.5, respectively, for 48 hrs. Percentage of infected cells (S11 Table) graphed. B, Violin plot of UPR and IFN module scores in HAP1 cells, split by challenge. C, Violin plot of module scores of UPR branches (ATF6, PERK, IRE1) in HAP1 cells, split by challenge. D, Feature plots of HAP1 cells. Features include: IFNB1, ISG15, IFI6, and IFITM1 gene expression in all HAP1 cells. E, Violin plot of IFN module scores in unchallenged HAP1 cells, grouped by guide detected. Line represents median value of module scores in non-target guide-detected cells. F, Feature scatter plots of HAP1 cells plotted by UPR module score and viral counts. Number in top right corner denotes Spearman’s correlation coefficient. (TIF) [file ppat.1014279.s021.tif]

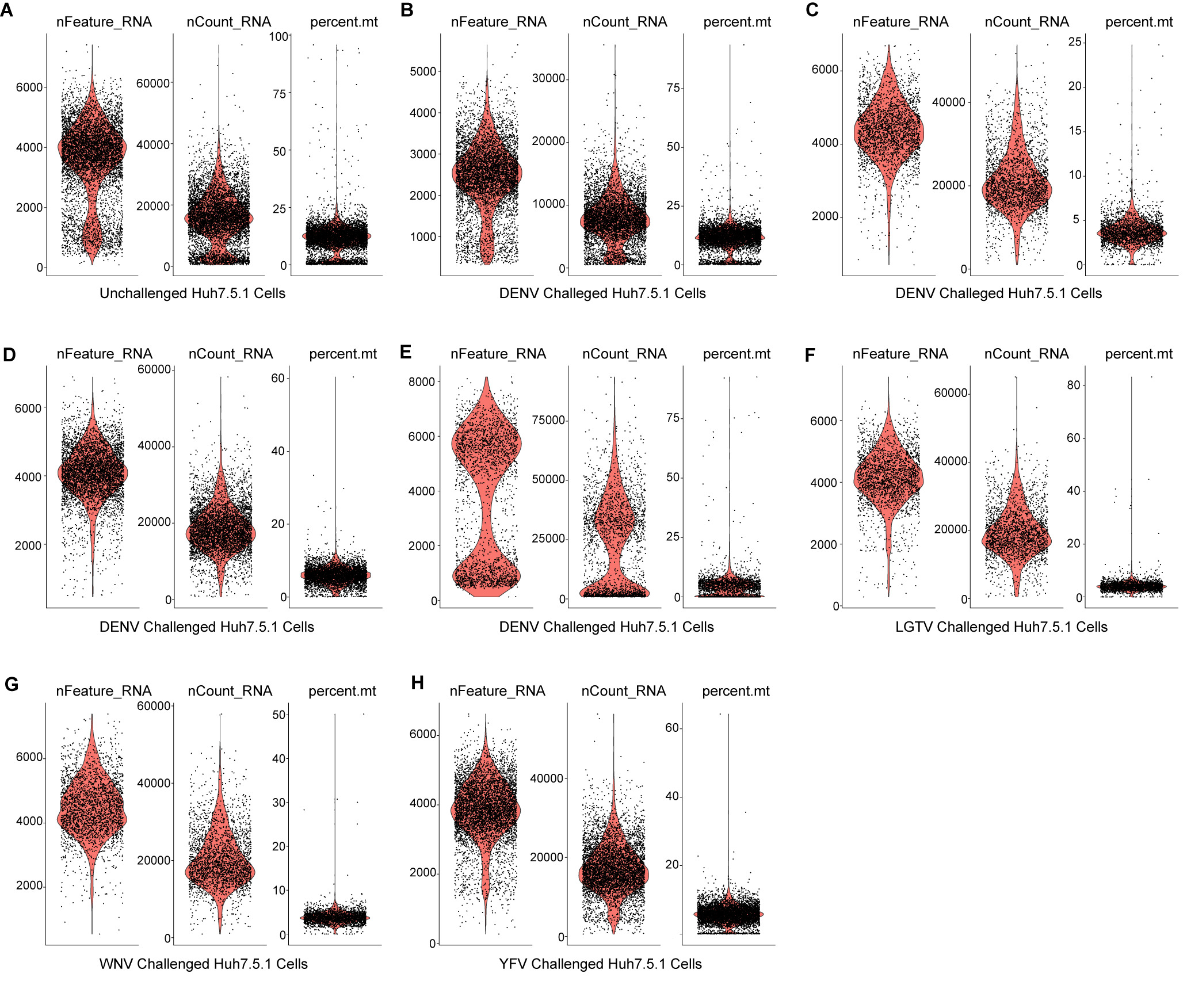

Supplement: S5 Fig — A-H, Violin plots for number of RNA features, counts of RNA, and percent mitochondrial genes. Cut offs were made according to each data set (see S15 Table for values used) before data sets were merged into final Seurat Object (SO). A, Unchallenged Huh7.5.1 cells. B, DENV-challenged Huh7.5.1 cells, biological replicate 1. C, DENV-challenged Huh7.5.1 cells, biological replicate 2. D, DENV-challenged Huh7.5.1 cells, biological replicate 3. E, DENV-challenged Huh7.5.1 cells, biological replicate 4. F, LGTV-challenged Huh7.5.1 cells. G, WNV-challenged Huh7.5.1 cells. H, YFV-challenged Huh7.5.1 cells. (TIF) [file ppat.1014279.s022.tif]

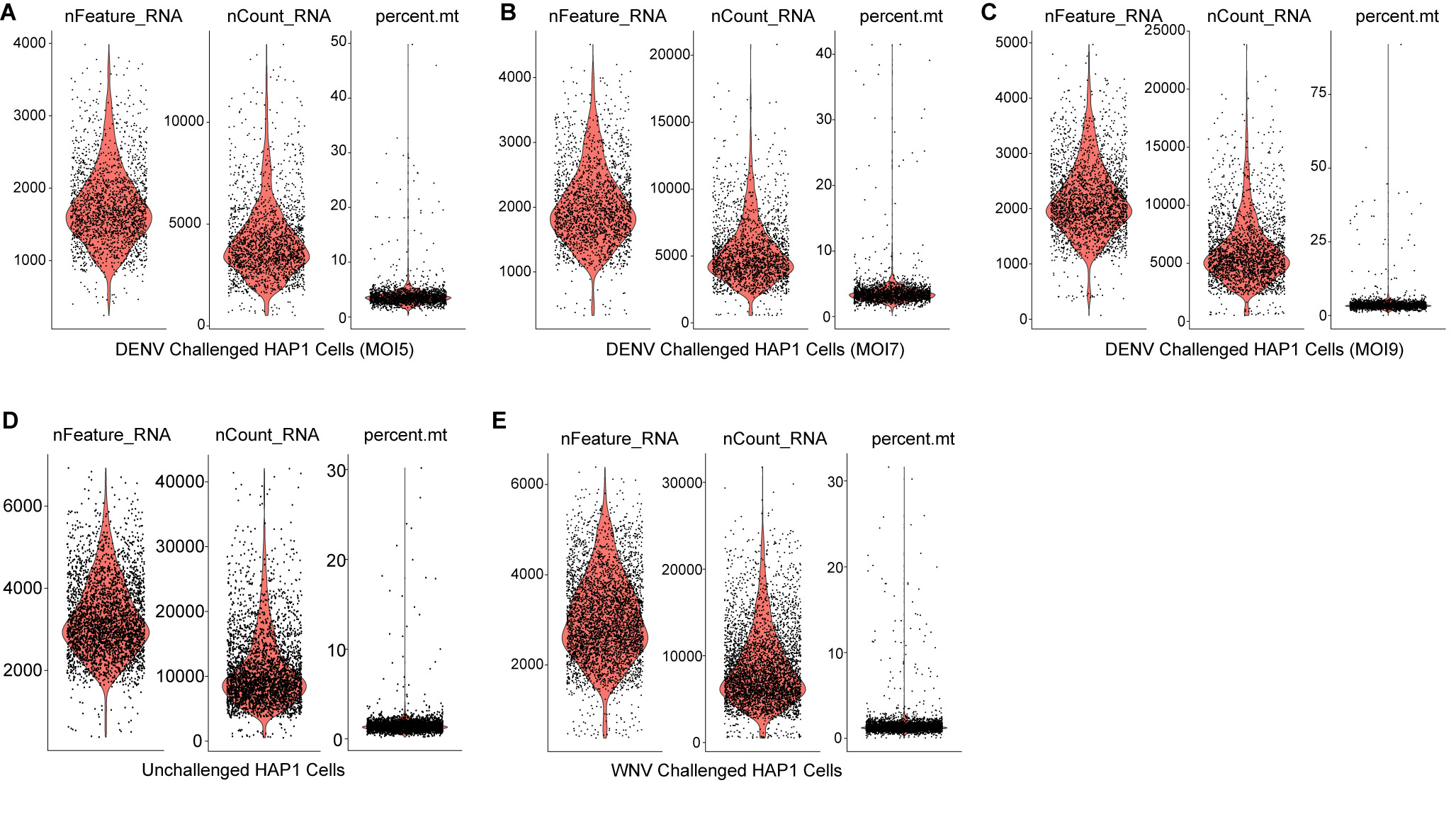

Supplement: S6 Fig — A-E, Violin plots for number of RNA features, counts of RNA, and percent mitochondrial genes. Cut offs were made according to each data set (S15 Table for values used) before data sets were merged into final Seurat Object (SO). A, DENV-challenged HAP1 cells (MOI 5). B, DENV-challenged HAP1 cells (MOI 7). C, DENV-challenged HAP1 cells (MOI 9). D, Unchallenged HAP1 cells. E, WNV-challenged HAP1 cells. (TIF) [file ppat.1014279.s023.tif]
