# Supplementary material for: Dissecting the host determinants of orthoflavivirus infection using QIC-seq
Source: PLoS Pathog. 2026 Jun 26;22(6):e1014279. doi: 10.1371/journal.ppat.1014279 (PMC13327515; doi:10.1371/journal.ppat.1014279)
Supplement: Attachment — Submitted filename: Rebutttal_Dupzyk_et_al_QIC_seq.pdf [file ppat.1014279.s024.pdf]

Editor's comment:

Peer reviewers were very positive about the quality of this study and the technical aspects of the research presented in this manuscript. However, Reviewer 3 notes some aspects of single-cell analysis that could be clarified by analyzing the existing data already presented in the manuscript, or whose experimental limitations should be noted.

We thank all reviewers and the editor for their helpful and insightful comments and suggestions in reviewing this manuscript. Based on the reviewer's comments, we have performed more in-depth analysis on the interferon response in HAP1 cells, distinguished the individual UPR branches, and explored whether individual gene knockouts affect transcriptional responses by performing differential gene expression in unchallenged and virus challenged cells. We believe that incorporating these changes have greatly improved the manuscript, and we thank reviewers again for their thoughtful comments.

Reviewer 1:

Reviewer #1: In this manuscript Dupzyk et al. present a new, integrative method of scRNA-seq to measure non-polyadenylated viral RNA, host cell transcriptome, and CRISPR gRNA all within the same cell. The ability to capture vRNA from non-polyadenlyated viruses and correlate with the host cell transcriptome in single cells is an exciting new direction and improvement on previous virus-inclusive scRNA-seq for flaviviruses. Additionally, the ability to measure virus replication in response to perturbation via CRISPR allows for a more nuanced analysis of host factor dependencies during viral infection. The authors validate their novel protocol across four different flaviviruses and in two cell types, lending credibility to this experimental system. They examine the impact of 20 different host factors on viral replication across 4 viruses and correlate host cell transcriptomics to viral replication in two different viruses. The manuscript is well written and very easy to follow, the figures are clear, and the validation for the technique is solid. I only have minor concerns and suggest additional characterization of infections in HAP1 cells could strengthen the manuscript by allowing for more precise interpretation of the data.

Reviewer 1 major issues:

Reviewer #1: No major issues. Additional characterization of viral infections in HAP1 cells would be extremely helpful in interpreting the data, but as the major contribution of this work is the development of a novel technique this is not necessary for the manuscript to add value to the field.

We thank the reviewer for their thorough review of the manuscript and the positive comments. We have addressed the reviewer's minor concerns and added additional characterization of infections in HAP1 cells.

Reviewer 1 minor issues:

Reviewer #1: • Line 162 the authors state "Generally, host factors important for DENV were also important for other orthoflaviviruses, suggesting a conserved role of these cellular factors in orthoflavivirus RNA replication". I would challenge this conclusion as there are only 2 factors of the tested 20 which significantly reduce infection of all 4 flaviviruses. And indeed the heading to this section is "Divergent orthoflaviviruses have dependencies on unique host factors". This is a minor point and may be differences in interpretations between the author and the reader.

We thank reviewer 1 for raising this valid point. This original wording emphasized the conserved nature of some of the host factors, while the comparison also revealed interesting differences. We changed the sentence to a more neutral statement "Our analysis revealed commonalities and differences in host factor utilization by the tested orthoflaviviruses (Fig. 2A-D)."

• Fig 3A: You note that as expected there is no interferon induction in Huh7.5.1 cells due to defects in viral

sensing in these cells, and as expected there is very little change in annotated ISGs. However, the authors highlight interferon stimulated genes in Fig 3 on multiple graphs. My understanding is that this is because the authors use this gene list in subsequent data analysis of HAP1 cell infections, however I find it seems out of place and is unnecessarily confusing. This is possibly a minor concern/reader preference.

We included the ISG analysis in Huh7.5.1 cells to emphasize the differences between Huh7.5.1 and HAP1 cells but can understand that this is unnecessarily confusing as there is little to no ISG activation. We have removed the color annotation of ISG's in Fig 3 and mentioning of the generation of the ISG master list. The latter is now introduced later, when analyzing the HAP1 infections.

• Figure 5: The data associated with this figure (and others) would be much easier to interpret with additional characterization of DENV infection in HAP1 cells and questions stem from lack of comprehensive characterization being presented. The authors provide a few interpretations of their data in the discussion, but hypotheses could be easily narrowed down by some additional experiments.

Fig 5A: It is unclear to me whether there is no IFN upregulation and thus no ISGs in DENV upregulated HAP1 cells, whether there is just no difference when stratifying across infection level as seen in WNV levels, or whether there is no ISG upregulation due to successful NS5 mediated blockade of STAT signaling (authors mention this in discussion). Thus, it would be nice to see global measurements of innate immune activation (phospho-IRF3, IFNB mRNA levels) at these different MOIs and timepoints for DENV and WNV infection in HAP1 cells.

We thank reviewer 1 for raising these points.

We agree that ISG upregulation might be missed when we would only rely on the stratifying approach e.g. because low level of infection already upregulate ISGs to the same extent as high level of infection or because, as in the case of WNV, paracrine effects are in play. However, we also see a lack of ISG upregulation when comparing the DENV-challenged sample to the unchallenged sample using the IFN module score (Supplemental figure 4B) and in the validation experiment (Fig 6D).

The other point raised is whether the lack of ISG upregulation could be due to successful NS5 blockade of STAT1 signaling by DENV. This is a possibility, but it is also possible that less type I interferon is produced e.g. because DENV is better at preventing PAMP recognition, which leads to initial interferon production mainly via RIG-I. It is not trivial to distinguish between the early response versus the late response as there is a positive feedback loop where interferon beta itself is an ISGs upregulated via paracrine STAT signaling. Prompted by the reviewer's valid point, we further looked at specific individual genes present in the IFN module and project them on the UMAP. We chose ISG15, IFI6, and IFITM1 as typical interferon stimulated genes resulting from STAT signaling and found that their pattern matched each other and the UPR module score. When assessing the interferon mRNA levels, interferon alpha or lambda mRNA levels were below the level of detection but we did observe cells with high levels of interferon beta (IFNB1) mRNA levels. Interestingly, this was a very small population of cells in contrast to the much larger population of cells found with the UPR module score and ISG15, IFI6, and IFITM1. We have added this analysis as a new panel (Supplemental 4D). This emphasizes the complexity and heterogeneity of IFN responses upon (flavi)viral infection. Single cell sequencing of SARS-CoV-2 infected cells similarly revealed that within a cell population only a small subsets of cells are IFN producers, while there is a much larger population that responds with ISG upregulation (PMID: 33585804).

The authors posit that it is possible DENV is not replicating to sufficient levels in HAP1 cells to induce innate immune activation – can you provide infection percentages, titers, etc. to measure relative infection of DENV and WNV in HAP1 cells? As above, is there data to show innate immune activation is not occurring instead of the alternative hypothesis of blockade of STAT signaling?

We thank reviewer 1 for this question and have now provided additional data from an experiment designed to compare viral infection percentages between DENV- and WNV -challenged HAP1 cells. HAP1 cells were challenged with DENV (MOI 7) or WNV (MOI 2.5) as used throughout the study, for 48 hours prior to staining

with the pan orthoflavivirus envelope antibody and analyzed by flow cytometry. Data is presented in Supplemental Figure 4A. At these MOIs, comparable levels of infection were observed for DENV and WNV, with 84% and 93% of cells infected, respectively. While we still cannot exclude that there are more subtle differences in replication between DENV and WNV, it is unlikely that reduced replication is solely responsible for the lack of ISG activation. We have changed the wording in the Discussion to convey that it is a less likely possibility.

It is not trivial to distinguish between whether innate immune activation is not occurring instead of the alternative hypothesis of blockade of STAT signaling given the cellular heterogeneity, the multiple mechanisms by which flaviviruses can evade both detection of the viral RNA and the secondary STAT signaling. Our data revealed a striking difference between DENV2 (16681 strain) and WNV (Kunjin strain) in the same cell type, which provides a starting point for detailed mechanistic studies, but we believe that this falls outside the scope of the current manuscript.

Figure S3: In the discussion the authors state "it is also possible that lower efficiency of [DENV] RNA replication compared to WNV infection contributed to the failure to trigger an IFN response." However, in Figure S3 the UMI counts look to be roughly the same between WNV and DENV. What do viral titers look like produced in these cells at these MOIs and timepoints? Or other measures quantifiable measures of viral replication. (Similar/related to comment above)

Please see response above.

Fig S3: DENV infections were performed at 3 different MOIs, do you see effects of these MOIs on viral RNA levels in cells as read out by UMI? For example, can you deconvolve the different MOIs in Supplemental figure 3A. This would be an interesting additional way of interpreting the data.

We thank reviewer 1 for this question. The slightly different MOIs were chosen after pilot experiments already narrowed down the MOI that leads to equivalent infection levels between DENV and WNV. Per the reviewer's suggestion we have deconvolved the different MOIs in Supplemental figure 3A and present this as new Supplemental figure 3C, 3D and 3E. We do not see readily apparent differences in viral RNA levels in cells as read out by UMI (e.g. compare the non-target control median value in Supplemental figure 3C, 3D and 3E). Moreover, no obvious differences in gene expression responses were noted (data not shown).

Figure 6: What is meant by two sets of 3 biological replicates? Is this not either 6 biological replicates or 2 sets of technical triplicates?

We thank reviewer 1 for drawing our attention to our confusing wording. In this context, a biological replicate indicates infections in separate wells of a tissue culture plate. cDNA isolated from each biological replicate is then measured more than one time using qPCR, which reflects the technical replicate. For each of the biological replicates, the average of the technical replicate values is first calculated, and the statistics are performed using this one average value per biological replicate. A "set" just means that the experiment (in biological replicate) was run on the same day. To avoid confusion, we have removed the color coding of the "sets" in figure 6 and just mention the number of biological and technical replicates.

Figure 6: What percentage of these cells are getting infected? Can you show non normalized data for the flow cytometry experiments? Relatedly, why did you choose to look at ISG15? Can you measure IFNB by qPCR? This would be additionally helpful in interpretation of Figure 5.

For both HAP1 and Huh7.5.1 cells, all non-normalized flow cytometry values are present in Supplemental Table 11. ISG15 is widely used as robust marker for interferon-stimulated gene activation upon IFNB treatment. We found it to be highly upregulated in the bystander cells alongside other typical ISGs such as IFI6 and IFITM1. Plotting ISG15, IFI6 and IFITM1 expression on the UMAP, reinforced the notion that ISG15 is suitable as representative ISG (Supplemental Fig 4B). After finding that IFNB1 was expressed only in a very small subpopulation of cells in the single cell data (Supplemental Fig 4B), we did not pursue IFNB qPCR as this signal was likely too low to be picked up by bulk RNA analysis.

Reviewer 2:

Reviewer #2: This study presents a method to quantify single cell host and viral transcriptional responses to infection in CRISPR-generated knock-out cells (QIC-Seq) and proceeds to conduct viral challenge experiments with DENV, WNV, YFV, and LGTV. Specifically, the CRISPR knockouts were targeted towards genes involved in polyprotein biogenesis and related to the ER membrane based on previous DENV studies (20 knockouts total). First, the paper reports on QIC-Seq on Huh 7.5.1 cells which are RIG-I deficient challenged with DENV. Second a QIC-Seq experiment with LGTV, WNV, and YFV was conducted to look at conserved factors, compared to DENV. Third, a pooled CRISPR approach was used to generate the cell library now in HAP1 cells that are IFN-signalling competent. This library was challenged with DENV at three (slightly) different MOIs and one WNV infection at a lower MOI. Finally, some QIC results were validated with qPCR.

The major result is that different orthoflaviviruses depend on different specific host factors (i.e. a knockout that resulted in strongly reduced viral replication in one virus would not affect other viruses as severely), although the host factors important for DENV were generally important in other flavis. Of note EMC6 knockout led to strong decreases in all four viruses and SSR3 in all but WNV. Subsequent experiments identified a positive correlation of the UPR cellular pathway with viral UMI counts. Finally, WNV challenge (but not DENV) showed strong activation of IFN stimulated genes and a negative correaltion with viral UMI counts.

Overall, this paper presents a promising single cell method to look at host and viral transcriptional responses to infections in CRISPR knockout cells. It is particularly noteworthy for its use of several different related viruses and two distinct cell lines. This paper will be a useful contribution to the host factor orthoflavivirus research field and to studies that examine viruses that have genomes lacking poly-A tails.

We thank the reviewer for their thorough review of the manuscript and the positive comments.

Reviewer 2 major issues:

1. The paper looks sound and the conclusions are appropriately constrained and supported by the data. Given that some of the results are confirmatory and that the approach is similar to others developed for other viruses (with the improvement being adapting for viral genomes w/o polyA tails), this manuscript would be greatly improved by highlighting the importance of the novel results in light of previous findings in the orthoflavivirus field and any potential general implications or applications of this methodology beyond the subfield.

We appreciate the comment to highlight the importance of the novel results in light of previous findings. We believe that the novelty is threefold and is highlighted in the manuscript. Firstly, while in other fields of biology Perturb-seq approaches are emerging, its applications in RNA virology are still scarce. The demonstration that the approach can be used to probe the effect of host factor knockdown to evaluate viral RNA replication and host response in single cells is technologically advanced. We believe that there is novelty in presenting the development of the approach, given that there is only a single other study that has reported a similar approach with an RNA virus of a different family (PMID: 37803001). Additionally, our approach is compatible with non-polyadenylated viruses including orthoflaviviruses, bunyaviruses and reoviruses. While orthoflaviviruses alone are already a large class of viruses of great medical importance, the two other families include additional viruses of clinical importance such as rotaviruses and Hantaviruses. Secondly, an important part of our study is demonstrating the utility of the approach to systematically compare the effect of CRISPR perturbation of host factors among multiple related viruses and between different cell types. The single prior study (PMID: 37803001) focused on one SARS-CoV-2 strain in one cell type. Thirdly, utilizing this approach, we uncovered commonalities and differences between the divergent flaviviruses in their dependencies on ER-proteostasis cellular factors. Our findings that West Nile and Langat virus are dependent on core components of the ER-associated degradation machinery, while

dengue and yellow fever virus are strictly dependent on subunits of the oligosaccharyltransferase complex suggest distinct interactions between divergent members of the same family.

Reviewer 2 minor concerns:

Line 230: Why were slightly different MOI's used? These probably should not be called biological replicates.

We thank reviewer 2 for this question. The slightly different MOIs were chosen after pilot experiments already narrowed down the MOI that leads to equivalent infection levels between DENV and WNV. Prompted by reviewer 1 and reviewer 2's comment, we have deconvolved the different MOIs in Supplemental figure 3A and present this as new Supplemental figure 3C, 3D and 3E. We do not see readily apparent differences in viral RNA levels in cells as read out by UMI (e.g. compare the non-target control median value in Supplemental figure 3C, 3D and 3E). Moreover, no obvious differences in gene expression responses were noted (data not shown). While we did not observe differences, we agree with the reviewer that we should avoid calling them biological replicate and we changed the text to reflect this.

Reviewer 3:

Reviewer #3: In this manuscript, Dupzyk and colleagues present a technically strong application of QIC-seq to compare host factor dependencies and transcriptional responses across orthoflaviviruses. The platform itself is well executed and adaptable, particularly for non-polyadenylated RNA viruses. However, the current biological scope limits the conceptual advance. The CRISPR library focuses on host factors that are already implicated in flavivirus infection, and the study largely confirms known dependencies (e.g., OST and ERAD components) and established transcriptional responses such as UPR activation. The latter in itself was not described in a global viral context even though the results would suggest UPR implications in multiple viruses, in this case, orthoflaviviruses. While the side-by-side comparison across viruses is useful, it did not yet yield sufficiently novel mechanistic insight to justify publication in its present form.
The single-cell approach offers substantial potential, but deeper interrogation of cellular heterogeneity, perturbation-specific transcriptional programs, or identification of previously unrecognized regulatory pathways would be necessary to elevate the impact. Expanding beyond a targeted gene set or incorporating functional dissection of UPR or IFN signaling pathways could significantly strengthen the work.

We thank the reviewer for their thorough review of the manuscript, and their mentioning of the strengths as technically strong, well executed and adaptable. We appreciate their request for additional characterization of the dataset, which we have now included.

Reviewer 3 major issues:

Reviewer #3: Major points to consider

1. Single-cell RNA-seq is often justified by its ability to uncover cellular heterogeneity, divergent infection states, rare cell populations, varying antiviral vs proviral transcriptional programs. However, in this study, the transcriptional response in Huh7.5.1 cells is largely dominated by UPR induction. Also, IFN activation is absent due to RIG-I deficiency as expected. Lastly, in HAP1 cells, the main insight is IFN upregulation in bystander cells during WNV infection, which aligns with known paracrine interferon dynamics. There is limited exploration of transcriptional heterogeneity beyond module scores and high/low infection categories. E.g., are there distinct subclusters within highly infected cells? Do certain knockouts shift the transcriptional trajectory beyond viral load effects?

We thank reviewer 3 for requesting additional exploratory data analysis on the mentioned topics.

To better characterize the effect of individual knockouts, we have now included additional differential gene expression of the gRNAs targeting genes versus the non-target control in infected and non-infected cells.

In Huh7.5.1 unchallenged cells, knockout of several genes including SEL1L, UBE2G2, and STT3A moderately increased the UPR module score compared to control, non-targeting sgRNAs (Fig. 3C bottom panel). In line with this, differential gene expression analysis showed that the widely used UPR marker HSPA5 (also named BiP/GPR78) was upregulated upon knockout of these genes, although overall few genes met the thresholds we set for log fold change, or adjusted p values. (Supplemental Table 5). DENV-challenge strongly increased the UPR module score as was apparent in cells containing the non-targeting sgRNAs (Fig. 3C upper panel). Cells containing sgRNAs targeting the host factors that most impacted DENV replication, including STT3A, STT3B and SSR3, displayed much reduced activation, indicating that DENV infection is the main driver of UPR activation. In agreement with this, cells containing sgRNAs targeting DENV-essential host factors showed a decreased expression in multiple UPR genes when compared to virally challenged non-target guide-detected cells (Supplemental Table 5).

This differential gene expression analysis is included in Supplemental Tables 5 and 10, for Huh7.5.1 infection and HAP1, respectively.

In terms of rare cell populations and antiviral responses, based on this and reviewer's #1 comment, we further looked at specific individual genes present in the IFN module and project them on the UMAP. We chose ISG15, IFI6, and IFITM1 as typical interferon stimulated genes resulting from STAT signaling and found that their pattern closely matched each other and the UPR module score. When assessing the interferon mRNA levels, interferon alpha or lambda mRNA levels were below the level of detection but we did observe cells with high levels of interferon beta (IFNB1) mRNA levels. Interestingly, this was a very small population of cells in contrast to the much larger population of cells found with the UPR module score and ISG15, IFI6, and IFITM1. We have added this analysis as a new panel (Supplemental 4D). This emphasizes the complexity and heterogeneity of IFN responses upon (flavi)viral infection. Single cell sequencing of SARS-CoV-2 infected cells similarly revealed that within a cell population only a small subsets of cells are IFN producers, while there is a much larger population responds via ISG upregulation infection (PMID: 33585804).

2. The manuscript emphasizes that UPR activation is a universal feature of orthoflavivirus infection. While UPR induction is clearly observed, this phenomenon has already been reported for DENV and other flaviviruses. The current work does not substantially explored which UPR branches (IRE1, PERK, ATF6) are most engaged, ar they correlated to viral transcript load, and if UPR activation functionally impacts replication. The claim of universal upregulation would make more sense if were linked mechanistically to differential host factor dependencies or to the viral replication efficiency or RNA count.

To address this in the manuscript we have included gene modules for the ATF6, PERK and IRE1 pathways (information gathered from the same publication used to generate the UPR module) and applied module scores to each cell. These data are added into figures Supplemental 2C, and Supplemental 4B and discussed in line 202-204 and 289-291. We have included the ATF6, PERK and IRE1 module lists in Supplemental table 7. Interestingly, while all tested virus activate the three UPR branches without obvious virus-specific differences, we found that, in HAP1 cells, WNV seems to activate the PERK pathway more than the other two branches.

As stated above, we found that the gRNA's most actively repressing replication, also prevented UPR activation suggesting that DENV infection drives the UPR response.

We thank reviewer 3 for the suggestion to include the three branches of UPR activation, which contributes to the better characterization of the data in the revised manuscript.

3. Though the study discusses IFN signaling in HAP1 cells, there is overall lack of exploring if IFN response correlates with specific knockouts, or if any ER-proteostasis genes modulate IFN induction, or if viral load-independent transcriptional programs (if any) emerge as well.

We thank reviewer 3 for this suggestion. To explore if specific knockouts could have a viral load independent effect on IFN signaling, we plot the IFN module score of unchallenged HAP1 cells by guides detected (now included as Supplemental Fig. 4e). We do not observe an increased baseline interferon response in any of the individual knockouts. We also explored if IFN response (or other genes) correlates with specific

knockouts upon viral challenge using differential gene expression comparing gRNAs targeting specific genes with the control, non-targeting gRNA. This analysis highlighted genes involved in amino-acid biosynthesis including ASS1, whose upregulation was prevented by gRNAs particularly active in repressing DENV replication. For WNV infection, we did not see differential gene expression between non-target and individual genes. This is presented in Supplemental table 10, and discussed in lines 275-299.

Reviewer 3: minor issues: none
