# Supplementary material for: Dissecting the host determinants of orthoflavivirus infection using QIC-seq
Source: PLoS Pathog. 2026 Jun 26;22(6):e1014279. doi: 10.1371/journal.ppat.1014279 (PMC13327515; doi:10.1371/journal.ppat.1014279)
Supplement: Peer review history

# Peer review: Decision Letter 0

**DOI:** 10.1371/journal.ppat.1014279.r001

**Authors:** Alexander E. Gorbalenya, Priya S Shah

4 Mar 2026

PPATHOGENS-D-26-00199

Dissecting the host determinants of orthoflavivirus infection using QIC-seq

PLOS Pathogens

Dear Dr. Carette,

Thank you for submitting your manuscript to PLOS Pathogens. After careful consideration, we feel that it has merit but does not fully meet PLOS Pathogens's publication criteria as it currently stands. Therefore, we invite you to submit a revised version of the manuscript that addresses the points raised during the review process.

Please submit your revised manuscript by May 03 2026 11:59PM. If you will need more time than this to complete your revisions, please reply to this message or contact the journal office at plospathogens@plos.org. When you're ready to submit your revision, log on to https://www.editorialmanager.com/ppathogens/ and select the 'Submissions Needing Revision' folder to locate your manuscript file.

Please include the following items when submitting your revised manuscript:

* A letter that responds to each point raised by the editor and reviewer(s). You should upload this letter as a separate file labeled 'Response to Reviewers'. This file does not need to include responses to any formatting updates and technical items listed in the 'Journal Requirements' section below.

* A marked-up copy of your manuscript that highlights changes made to the original version. You should upload this as a separate file labeled 'Revised Manuscript with Track Changes'.

* An unmarked version of your revised paper without tracked changes. You should upload this as a separate file labeled 'Manuscript'.

If you would like to make changes to your financial disclosure, competing interests statement, or data availability statement, please make these updates within the submission form at the time of resubmission. Guidelines for resubmitting your figure files are available below the reviewer comments at the end of this letter.

We look forward to receiving your revised manuscript.

Kind regards,

Priya S Shah

Guest Editor

PLOS Pathogens

Alexander Gorbalenya

Section Editor

PLOS Pathogens

Sumita Bhaduri-McIntosh

Editor-in-Chief

PLOS Pathogens

orcid.org/0000-0003-2946-9497

Michael Malim

Editor-in-Chief

PLOS Pathogens

orcid.org/0000-0002-7699-2064

**Additional Editor Comments:**

Peer reviewers were very positive about the quality of this study and the technical aspects of the research presented in this manuscript. However, Reviewer 3 notes some aspects of single-cell analysis that could be clarified by analyzing the existing data already presented in the manuscript, or whose experimental limitations should be noted.

**Journal Requirements:**

1) Please ensure that the CRediT author contributions listed for every co-author are completed accurately and in full.

At this stage, the following Authors/Authors require contributions: Allison Dupzyk, Benjamin Waldman, James Zengel, Fabio Zanini, and Jan Carette. Please ensure that the full contributions of each author are acknowledged in the "Add/Edit/Remove Authors" section of our submission form.

The list of CRediT author contributions may be found here: https://journals.plos.org/plospathogens/s/authorship#loc-author-contributions

2) We ask that a manuscript source file is provided at Revision. Please upload your manuscript file as a .doc, .docx, .rtf or .tex. If you are providing a .tex file, please upload it under the item type u2018LaTeX Source Fileu2019 and leave your .pdf version as the item type u2018Manuscriptu2019.

3) Please upload all main figures as separate Figure files in .tif or .eps format. For more information about how to convert and format your figure files please see our guidelines:

https://journals.plos.org/plospathogens/s/figures

4) We have noticed that you have uploaded Supporting Information files, but you have not included a list of legends. Please add a full list of legends for your Supporting Information files after the references list.

**Reviewers' Comments:**

Reviewer's Responses to Questions

**Part I - Summary**

Please use this section to discuss strengths/weaknesses of study, novelty/significance, general execution and scholarship.

Reviewer #1: In this manuscript Dupzyk et al. present a new, integrative method of scRNA-seq to measure non-polyadenylated viral RNA, host cell transcriptome, and CRISPR gRNA all within the same cell. The ability to capture vRNA from non-polyadenlyated viruses and correlate with the host cell transcriptome in single cells is an exciting new direction and improvement on previous virus-inclusive scRNA-seq for flaviviruses. Additionally, the ability to measure virus replication in response to perturbation via CRISPR allows for a more nuanced analysis of host factor dependencies during viral infection. The authors validate their novel protocol across four different flaviviruses and in two cell types, lending credibility to this experimental system. They examine the impact of 20 different host factors on viral replication across 4 viruses and correlate host cell transcriptomics to viral replication in two different viruses. The manuscript is well written and very easy to follow, the figures are clear, and the validation for the technique is solid. I only have minor concerns and suggest additional characterization of infections in HAP1 cells could strengthen the manuscript by allowing for more precise interpretation of the data.

Reviewer #2: This study presents a method to quantify single cell host and viral transcriptional responses to infection in CRISPR-generated knock-out cells (QIC-Seq) and proceeds to conduct viral challenge experiments with DENV, WNV, YFV, and LGTV. Specifically, the CRISPR knockouts were targeted towards genes involved in polyprotein biogenesis and related to the ER membrane based on previous DENV studies (20 knockouts total). First, the paper reports on QIC-Seq on Huh 7.5.1 cells which are RIG-I deficient challenged with DENV. Second a QIC-Seq experiment with LGTV, WNV, and YFV was conducted to look at conserved factors, compared to DENV. Third, a pooled CRISPR approach was used to generate the cell library now in HAP1 cells that are IFN-signalling competent. This library was challenged with DENV at three (slightly) different MOIs and one WNV infection at a lower MOI. Finally, some QIC results were validated with qPCR.

The major result is that different orthoflaviviruses depend on different specific host factors (i.e. a knockout that resulted in strongly reduced viral replication in one virus would not affect other viruses as severely), although the host factors important for DENV were generally important in other flavis. Of note EMC6 knockout led to strong decreases in all four viruses and SSR3 in all but WNV. Subsequent experiments identified a positive correlation of the UPR cellular pathway with viral UMI counts. Finally, WNV challenge (but not DENV) showed strong activation of IFN stimulated genes and a negative correaltion with viral UMI counts.

Overall, this paper presents a promising single cell method to look at host and viral transcriptional responses to infections in CRISPR knockout cells. It is particularly noteworthy for its use of several different related viruses and two distinct cell lines. This paper will be a useful contribution to the host factor orthoflavivirus research field and to studies that examine viruses that have genomes lacking poly-A tails.

Reviewer #3: In this manuscript, Dupzyk and colleagues present a technically strong application of QIC-seq to compare host factor dependencies and transcriptional responses across orthoflaviviruses. The platform itself is well executed and adaptable, particularly for non-polyadenylated RNA viruses.

However, the current biological scope limits the conceptual advance. The CRISPR library focuses on host factors that are already implicated in flavivirus infection, and the study largely confirms known dependencies (e.g., OST and ERAD components) and established transcriptional responses such as UPR activation. The latter in itself was not described in a global viral context even though the results would suggest UPR implications in multiple viruses, in this case, orthoflaviviruses. While the side-by-side comparison across viruses is useful, it did not yet yield sufficiently novel mechanistic insight to justify publication in its present form.

The single-cell approach offers substantial potential, but deeper interrogation of cellular heterogeneity, perturbation-specific transcriptional programs, or identification of previously unrecognized regulatory pathways would be necessary to elevate the impact. Expanding beyond a targeted gene set or incorporating functional dissection of UPR or IFN signaling pathways could significantly strengthen the work.

**********

**Part II – Major Issues: Key Experiments Required for Acceptance**

Please use this section to detail the key new experiments or modifications of existing experiments that should be absolutely required to validate study conclusions.

Generally, there should be no more than 3 such required experiments or major modifications for a "Major Revision" recommendation. If more than 3 experiments are necessary to validate the study conclusions, then you are encouraged to recommend "Reject".

Reviewer #1: No major issues. Additional characterization of viral infections in HAP1 cells would be extremely helpful in interpreting the data, but as the major contribution of this work is the development of a novel technique this is not necessary for the manuscript to add value to the field.

Reviewer #2: Major Comments

1. The paper looks sound and the conclusions are appropriately constrained and supported by the data. Given that some of the results are confirmatory and that the approach is similar to others developed for other viruses (with the improvement being adapting for viral genomes w/o polyA tails), this manuscript would be greatly improved by highlighting the importance of the novel results in light of previous findings in the orthoflavivirus field and any potential general implications or applications of this methodology beyond the subfield.

Reviewer #3: Major points to consider

1. Single-cell RNA-seq is often justified by its ability to uncover cellular heterogeneity, divergent infection states, rare cell populations, varying antiviral vs proviral transcriptional programs. However, in this study, the transcriptional response in Huh7.5.1 cells is largely dominated by UPR induction. Also, IFN activation is absent due to RIG-I deficiency as expected. Lastly, in HAP1 cells, the main insight is IFN upregulation in bystander cells during WNV infection, which aligns with known paracrine interferon dynamics. There is limited exploration of transcriptional heterogeneity beyond module scores and high/low infection categories. E.g., are there distinct subclusters within highly infected cells? Do certain knockouts shift the transcriptional trajectory beyond viral load effects?

2. The manuscript emphasizes that UPR activation is a universal feature of orthoflavivirus infection. While UPR induction is clearly observed, this phenomenon has already been reported for DENV and other flaviviruses. The current work does not substantially explored which UPR branches (IRE1, PERK, ATF6) are most engaged, ar they correlated to viral transcript load, and if UPR activation functionally impacts replication. The claim of universal upregulation would make more sense if were linked mechanistically to differential host factor dependencies or to the viral replication efficiency or RNA count.

3. Though the study discusses IFN signaling in HAP1 cells, there is overall lack of exploring if IFN response correlates with specific knockouts, or if any ER-proteostasis genes modulate IFN induction, or if viral load-independent transcriptional programs (if any) emerge as well.

**********

**Part III – Minor Issues: Editorial and Data Presentation Modifications**

Please use this section for editorial suggestions as well as relatively minor modifications of existing data that would enhance clarity.

Reviewer #1: • Line 162 the authors state “Generally, host factors important for DENV were also important for other orthoflaviviruses, suggesting a conserved role of these cellular factors in orthoflavivirus RNA replication”. I would challenge this conclusion as there are only 2 factors of the tested 20 which significantly reduce infection of all 4 flaviviruses. And indeed the heading to this section is “Divergent orthoflaviviruses have dependencies on unique host factors”. This is a minor point and may be differences in interpretations between the author and the reader.

• Fig 3A: You note that as expected there is no interferon induction in Huh7.5.1 cells due to defects in viral sensing in these cells, and as expected there is very little change in annotated ISGs. However, the authors highlight interferon stimulated genes in Fig 3 on multiple graphs. My understanding is that this is because the authors use this gene list in subsequent data analysis of HAP1 cell infections, however I find it seems out of place and is unnecessarily confusing. This is possibly a minor concern/reader preference.

• Figure 5: The data associated with this figure (and others) would be much easier to interpret with additional characterization of DENV infection in HAP1 cells and questions stem from lack of comprehensive characterization being presented. The authors provide a few interpretations of their data in the discussion, but hypotheses could be easily narrowed down by some additional experiments.

oFig 5A: It is unclear to me whether there is no IFN upregulation and thus no ISGs in DENV upregulated HAP1 cells, whether there is just no difference when stratifying across infection level as seen in WNV levels, or whether there is no ISG upregulation due to successful NS5 mediated blockade of STAT signaling (authors mention this in discussion). Thus, it would be nice to see global measurements of innate immune activation (phospho-IRF3, IFNB mRNA levels) at these different MOIs and timepoints for DENV and WNV infection in HAP1 cells.

oThe authors posit that it is possible DENV is not replicating to sufficient levels in HAP1 cells to induce innate immune activation – can you provide infection percentages, titers, etc. to measure relative infection of DENV and WNV in HAP1 cells? As above, is there data to show innate immune activation is not occurring instead of the alternative hypothesis of blockade of STAT signaling?

•Figure S3: In the discussion the authors state “it is also possible that lower efficiency of [DENV] RNA replication compared to WNV infection contributed to the failure to trigger an IFN response.” However, in Figure S3 the UMI counts look to be roughly the same between WNV and DENV. What do viral titers look like produced in these cells at these MOIs and timepoints? Or other measures quantifiable measures of viral replication. (Similar/related to comment above)

•Fig S3: DENV infections were performed at 3 different MOIs, do you see effects of these MOIs on viral RNA levels in cells as read out by UMI? For example, can you deconvolve the different MOIs in Supplemental figure 3A. This would be an interesting additional way of interpreting the data.

•Figure 6: What is meant by two sets of 3 biological replicates? Is this not either 6 biological replicates or 2 sets of technical triplicates?

•Figure 6: What percentage of these cells are getting infected? Can you show non normalized data for the flow cytometry experiments? Relatedly, why did you choose to look at ISG15? Can you measure IFNB by qPCR? This would be additionally helpful in interpretation of Figure 5.

Reviewer #2: Minor Comments

Line 230: Why were slightly different MOI's used? These probably should not be called biological replicates.

Reviewer #3: (No Response)

**********

PLOS authors have the option to publish the peer review history of their article (what does this mean?). If published, this will include your full peer review and any attached files.

If you choose “no”, your identity will remain anonymous but your review may still be made public.

**Do you want your identity to be public for this peer review?**  For information about this choice, including consent withdrawal, please see our Privacy Policy.

Reviewer #1: No

Reviewer #2: No

Reviewer #3: No

[NOTE: If reviewer comments were submitted as an attachment file, they will be attached to this email and accessible via the submission site. Please log into your account, locate the manuscript record, and check for the action link "View Attachments". If this link does not appear, there are no attachment files.]

**Figure resubmission:**

-->While revising your submission, we strongly recommend that you use PLOS’s NAAS tool (https://ngplosjournals.pagemajik.ai/artanalysis) to test your figure files. NAAS can convert your figure files to the TIFF file type and meet basic requirements (such as print size, resolution), or provide you with a report on issues that do not meet our requirements and that NAAS cannot fix.

After uploading your figures to PLOS’s NAAS tool - https://ngplosjournals.pagemajik.ai/artanalysis, NAAS will process the files provided and display the results in the "Uploaded Files" section of the page as the processing is complete. If the uploaded figures meet our requirements (or NAAS is able to fix the files to meet our requirements), the figure will be marked as "fixed" above. If NAAS is unable to fix the files, a red "failed" label will appear above. When NAAS has confirmed that the figure files meet our requirements, please download the file via the download option, and include these NAAS processed figure files when submitting your revised manuscript.

**Reproducibility:**

To enhance the reproducibility of your results, we recommend that authors of applicable studies deposit laboratory protocols in protocols.io, where a protocol can be assigned its own identifier (DOI) such that it can be cited independently in the future. Additionally, PLOS ONE offers an option to publish peer-reviewed clinical study protocols. Read more information on sharing protocols at https://plos.org/protocols?utm_medium=editorial-email&utm_source=authorletters&utm_campaign=protocols

# Author comment: Author response to Decision Letter 1

**DOI:** 10.1371/journal.ppat.1014279.r002

24 Apr 2026

Attachment Submitted filename: Rebutttal_Dupzyk_et_al_QIC_seq.pdf

# Peer review: Decision Letter 1

**DOI:** 10.1371/journal.ppat.1014279.r003

**Authors:** Alexander E. Gorbalenya, Priya S Shah

18 May 2026

Dear Carette,

We are pleased to inform you that your manuscript 'Dissecting the host determinants of orthoflavivirus infection using QIC-seq' has been provisionally accepted for publication in PLOS Pathogens.

Before your manuscript can be formally accepted you will need to complete some formatting changes, which you will receive in a follow up email. A member of our team will be in touch with a set of requests.

Please note that your manuscript will not be scheduled for publication until you have made the required changes, so a swift response is appreciated.

IMPORTANT: The editorial review process is now complete. PLOS will only permit corrections to spelling, formatting or significant scientific errors from this point onwards. Requests for major changes, or any which affect the scientific understanding of your work, will cause delays to the publication date of your manuscript.

Should you, your institution's press office or the journal office choose to press release your paper, you will automatically be opted out of early publication. We ask that you notify us now if you or your institution is planning to press release the article. All press must be co-ordinated with PLOS.

Thank you again for supporting Open Access publishing; we are looking forward to publishing your work in PLOS Pathogens.

Best regards,

Priya S Shah

Guest Editor

PLOS Pathogens

Alexander Gorbalenya

Section Editor

PLOS Pathogens

Sumita Bhaduri-McIntosh

Editor-in-Chief

PLOS Pathogens

orcid.org/0000-0003-2946-9497

Michael Malim

Editor-in-Chief

PLOS Pathogens

orcid.org/0000-0002-7699-2064

***********************************************************

Reviewer Comments (if any, and for reference):

Reviewer's Responses to Questions

**Part I - Summary**

Please use this section to discuss strengths/weaknesses of study, novelty/significance, general execution and scholarship.

Reviewer #1: I had no major concerns with the original submission. The authors addressed some of my minor concerns. I think there could have been better characterization of innate immune activation in HAP1 cells as suggested (looking at phosphorylation of IRF3 vs phosphorylation of STAT1 and time course kinetics of infection), but again this is not the point of the paper and I am satisfied with the data supporting the development of a novel protocol.

Reviewer #2: The revised paper goes above and beyond in response to reviewer comments. I especially want to highlight the significant effort that went into the in-depth characterization of HPA1 cells and the individual gene knockout experiments. The response to reviewers was thoughtful and included corresponding modifications. I particularly appreciated that the authors took the time to highlight the contributions of this study for a broader readership. It is an even more compelling contribution given these substantial changes.

Reviewer #3: The authors have answered the queries to my earlier points

**********

**Part II – Major Issues: Key Experiments Required for Acceptance**

Please use this section to detail the key new experiments or modifications of existing experiments that should be absolutely required to validate study conclusions.

Generally, there should be no more than 3 such required experiments or major modifications for a "Major Revision" recommendation. If more than 3 experiments are necessary to validate the study conclusions, then you are encouraged to recommend "Reject".

Reviewer #1: None.

Reviewer #2: (No Response)

Reviewer #3: The authors have answered the queries to my earlier points

**********

**Part III – Minor Issues: Editorial and Data Presentation Modifications**

Please use this section for editorial suggestions as well as relatively minor modifications of existing data that would enhance clarity.

Reviewer #1: None.

Reviewer #2: (No Response)

Reviewer #3: The authors have answered the queries to my earlier points

**********

PLOS authors have the option to publish the peer review history of their article (what does this mean?). If published, this will include your full peer review and any attached files.

If you choose “no”, your identity will remain anonymous but your review may still be made public.

**Do you want your identity to be public for this peer review?**  For information about this choice, including consent withdrawal, please see our Privacy Policy.

Reviewer #1: No

Reviewer #2: No

Reviewer #3: No

# Editor report: Acceptance letter

**DOI:** 10.1371/journal.ppat.1014279.r004

**Authors:** Alexander E. Gorbalenya, Priya S Shah

Dear Carette,

We are delighted to inform you that your manuscript, "Dissecting the host determinants of orthoflavivirus infection using QIC-seq," has been formally accepted for publication in PLOS Pathogens.

We have now passed your article onto the PLOS Production Department who will complete the rest of the pre-publication process. All authors will receive a confirmation email upon publication.

The corresponding author will soon be receiving a typeset proof for review, to ensure errors have not been introduced during production. Please review the PDF proof of your manuscript carefully, as this is the last chance to correct any scientific or type-setting errors. Please note that major changes, or those which affect the scientific understanding of the work, will likely cause delays to the publication date of your manuscript. Note: Proofs for Front Matter articles (Pearls, Reviews, Opinions, etc...) are generated on a different schedule and may not be made available as quickly.

Soon after your final files are uploaded, the early version of your manuscript, if you opted to have an early version of your article, will be published online. The date of the early version will be your article's publication date. The final article will be published to the same URL, and all versions of the paper will be accessible to readers.

For Research Articles, you will receive an invoice from PLOS for your publication fee after your manuscript has reached the completed accept phase. If you receive an email requesting payment before acceptance or for any other service, this may be a phishing scheme. Learn how to identify phishing emails and protect your accounts at https://explore.plos.org/phishing.

Thank you again for supporting open-access publishing; we are looking forward to publishing your work in PLOS Pathogens.

Best regards,

Sumita Bhaduri-McIntosh

Editor-in-Chief

PLOS Pathogens

orcid.org/0000-0003-2946-9497

Michael Malim

Editor-in-Chief

PLOS Pathogens

orcid.org/0000-0002-7699-2064
